# Effectiveness of telephone-based interventions for managing osteoarthritis and spinal pain: a systematic review and meta-analysis

**DOI:** 10.7717/peerj.5846

**Published:** 2018-10-30

**Authors:** Kate M. O’Brien, Rebecca K. Hodder, John Wiggers, Amanda Williams, Elizabeth Campbell, Luke Wolfenden, Sze Lin Yoong, Flora Tzelepis, Steven J. Kamper, Christopher M. Williams

**Affiliations:** 1 Hunter New England Population Health, Wallsend, NSW, Australia; 2 School of Medicine and Public Health, Hunter Medical Research Institute, University of Newcastle, Newcastle, NSW, Australia; 3 Centre for Pain, Health and Lifestyle, Ourimbah, NSW, Australia; 4 School of Public Health, University of Sydney, Sydney, NSW, Australia

**Keywords:** Systematic review, Meta-analysis, Osteoarthritis, Spinal pain, Telephone, Intervention, Pain, Disability

## Abstract

**Background:**

Osteoarthritis and spinal pain are common and burdensome conditions; however, the majority of patients with these conditions do not receive care that is consistent with clinical practice guidelines. Telehealth models of care have the potential to improve care for osteoarthritis and spinal pain patients. The aim of this review was to assess the effectiveness of verbal real-time telehealth interventions, including telephone-based and videoconferencing interventions to reduce pain intensity and disability in patients with osteoarthritis of the knee or hip and spinal pain (back or neck pain).

**Methods:**

We searched seven electronic databases from inception to May 2018. Randomised controlled trials (RCTs), cluster-RCTs, and non-randomised controlled trials were included. Two review authors independently extracted data for each included study. Primary outcomes were pain intensity and disability. We conducted primary meta-analyses combining all conditions with similar interventions and comparators. Standardised mean difference (SMD) and 95% confidence intervals (CIs) were calculated using random effects models. We used the Cochrane Risk of Bias tool to assess risk of bias, and GRADE to evaluate the quality of evidence.

**Results:**

We included 23 studies with 56 trial arms and 4,994 participants. All studies utilised telephone-based interventions. Only two studies used a telephone only approach and the remainder included educational materials and/or face-to-face components. We found no studies utilising videoconferencing. Meta-analysis showed telephone-based interventions (with educational materials) for osteoarthritis and spinal pain improved pain intensity (*n* = 5 trials, *n* = 1,357 participants, SMD −0.27, 95% CI [−0.53, −0.01], Tau^2^ = 0.06, *I*^2^ = 74%; moderate-quality evidence) and disability (*n* = 7 trials, *n* = 1,537 participants, SMD −0.21, 95% CI [−0.40, −0.02], Tau^2^ = 0.03, *I*^2^ = 56%; moderate-quality evidence) compared to usual care. Meta-analyses found telephone with face-to-face interventions does not improve pain and disability compared to usual care or face-to-face care alone.

**Discussion:**

We are moderately confident that telephone-based interventions reduce pain intensity and disability in patients with osteoarthritis and spinal pain compared to usual care, but telephone plus face-to-face interventions are no more effective than usual care or face-to-face interventions alone.

## Introduction

Internationally there is an emphasis on how to improve access, quality, and cost-effectiveness of health care (McLean et al., 2013). Faced with aging populations and increasing burden of lifestyle-related disease, health care systems require scalable solutions within their constrained resources to improve the health of populations (WHO, 2014). Over the past two decades, telehealth models of care have become a strategic priority in many governments with the aim of preventing and reducing the impact of chronic non-communicable diseases (McLean et al., 2013). Telehealth is defined as ‘the use of telecommunication technologies for the purpose of providing telemedicine (clinical care by health care profession), medical education, and health education over a distance’ (Australian Government Department of Health and Ageing, 2015). Telecommunication technologies include synchronous verbal communication (i.e. delivered via real-time) such as telephone calls and videoconferencing, and asynchronous non-verbal communication such as SMS, mobile applications, web-based programs, and email (McLean et al., 2013).

Osteoarthritis of the knee and hip, and back and neck pain (spinal pain) are the most burdensome musculoskeletal conditions (Hay et al., 2017; Vos et al., 2017), for which telehealth models of care have the potential to improve care and reduce costs (McLean et al., 2013; Dinesen et al., 2016). These conditions cause considerable disability, accounting for 12.8% of years lived with disability in 2016 worldwide (Vos et al., 2017), and they are responsible for substantial health care and social costs, which in western countries is estimated at 1–2.5% of the gross national product (March & Bachmeier, 1997). Additionally, osteoarthritis and spinal pain are poorly managed in routine care with the vast majority of patients not receiving care that is consistent with clinical practice guideline recommendations (Williams et al., 2010; Hunter, 2011; Brand et al., 2014).

Clinicians report many barriers to delivering recommended care for osteoarthritis and spinal pain (Ackerman, Buchbinder & Osborne, 2013). These include challenges related to limited consultation time, space, cost, and resources to deliver recommended care (Ackerman, Buchbinder & Osborne, 2013). Patients report facing barriers to accessing or attending care as result of geographical location or local service availability, travel time and transport requirements, work commitments, and ability to attend multiple appointments (March et al., 2010; Ackerman, Buchbinder & Osborne, 2013). Telehealth approaches overcome such barriers by providing better access to recommended care (McLean et al., 2013).

Telephone-based and videoconferencing interventions are a subset of telehealth models of care, which offer direct verbal patient-provider contact and have increasing appeal to support patient care via remote delivery. A previous review of telehealth interventions for patients with chronic non-specific low back pain, which included mostly asynchronous delivery via web-based platforms such as websites, email, and peer communication services, found these to be no more effective than minimalist interventions (i.e. health or non-health-related information) in improving pain and disability in these patients (Dario et al., 2017). In contrast, another recent review found utilising telephone-based and videoconferencing interventions improves physical function in post-surgical rehabilitation of musculoskeletal conditions, including spinal pain, rheumatoid arthritis, and osteoarthritis (Cottrell et al., 2017a). These results combined with other data revealing telephone-based models of care are preferred by patients with chronic musculoskeletal conditions (Williams et al., 2014; Cottrell et al., 2017b), suggest remotely delivered verbal real-time interventions such as by telephone or videoconferencing have promising effects. However, while a number of trials investigating the use of telephone-based interventions for osteoarthritis and spinal pain have been conducted (Weinberger et al., 1989; Allen et al., 2016; Bennell et al., 2017; Gialanella et al., 2017) there has been no comprehensive review of the evidence regarding the effectiveness of telephone-based or videoconferencing interventions for this patient group. As such, the primary objective of this systematic review was to assess the effectiveness of verbal real-time telehealth interventions, including telephone-based and videoconferencing interventions, to reduce pain intensity and disability in patients with osteoarthritis of the knee or hip and spinal pain (back or neck pain), compared to usual care or face-to-face interventions.

## Methods

### Search strategy

We conducted a systematic review following the PRISMA statement (Moher et al., 2009) and prospectively registered on PROSPERO (CRD42015027626). We searched Medline, Embase, AMED, Medline In-Process, PsycINFO, CINAHL, SportDiscus from inception to May 2018 to identify eligible studies (Table S1. Example search strategy for MEDLINE). We used a combination of relevant keywords based on those used in other systematic reviews to construct a search strategy including search terms for participants, intervention, study design, and comparator. The search strategy was reviewed and performed by an information specialist Debbie Booth (DB), and modified to suit each database. We searched trial registries (ClinicalTrials.gov, the Australian and New Zealand Clinical Trials Registry and the World Health Organisation International Clinical Trials Registry Platform) in May 2018. We also conducted a manual search of the reference lists of all included studies. The corresponding authors of all included studies were contacted via email to request details of any other potentially eligible studies.

### Study selection

We included randomised controlled trials (RCTs), cluster RCTs (C-RCTs) and non-randomised controlled trials that had a parallel comparison group as per the a priori trial registration. Trials with non-random assignment of groups were included given Medical Research Council recommendations that non-randomised designs may represent an appropriate evaluation design for some complex health promotion interventions (Craig et al., 2008). Eligible comparison groups included other interventions, no treatment, usual care, wait-list control or attention control. To be eligible, trials had to include participants with osteoarthritis of the knee or hip, or spinal pain (back or neck pain). We included trials that defined osteoarthritis as confirmed by clinical assessment or medical diagnosis, including patient self-report of such diagnosis, with or without diagnostic imaging. For spinal pain, we included any trial that included clinically diagnosed or participant self-reported back, neck, thoracic, or cervical pain. Studies with mixed populations of musculoskeletal conditions were included where separate data were provided for osteoarthritis and spinal pain. We included trials that did not specify the location of osteoarthritis, as we assumed those studies would be representative of patients with knee or hip osteoarthritis as these are the most prevalent types of osteoarthritis (Vos et al., 2016). There were no restrictions on intensity or duration of participant symptoms. Studies that included patients with a serious pathology (e.g. cancer, infection, etc.) or included patients in the postoperative period were excluded. We excluded studies including other chronic pain conditions such as headache, rheumatoid arthritis, and neuropathic pain because they have a clearly different etiology and clinical course. There were no restrictions on the basis of publication language, status or date.

We included trials that involved service delivery by any person (i.e. therapist, health professional or trained operator) by telephone or videoconferencing in which there was a direct person-to-person verbal exchange of information. The service could be used to provide any aspect of care (e.g. delivery of advice, education, behaviour modification treatment, ongoing support). We included studies that specifically aimed to test the effectiveness of a telephone-based or videoconferencing intervention. Complex interventions with one or more delivery component (e.g. face-to-face sessions or educational materials in addition to telephone or videoconferencing) were included if the telephone or videoconferencing component was the main method of intervention delivery, defined as at least 50% of the total number of intervention contacts conducted via telephone or videoconferencing. Trials were included if they reported a valid measure of at least one of the following primary review outcomes: pain intensity or disability (including physical function), the core outcomes recommended to be used in clinical trials (e.g. OMERACT-OARSI, IMMPACT) (Dworkin et al., 2005). Secondary outcomes included psychological symptoms, self-efficacy, behavioural outcomes related to treatment (weight loss, physical activity, healthcare or medication use, treatment adherence), health-related quality of life, recovery, subjective improvement in symptoms, fear avoidance, and adverse events.

### Data extraction and quality assessment

After removing duplicates, two pairs of independent authors screened the titles, abstracts, and full-texts of all identified studies (KO and AW, RH, LW, SY, SK) (Higgins & Green, 2011). A third reviewer resolved any disagreements (CW). Two authors independently extracted data from eligible studies, using a standardised data extraction tool (KO and AW). Information regarding study characteristics (design, participants, interventions, outcomes) was extracted and a third author resolved any disagreement (CW). Authors were contacted to provide further information if eligibility was unclear or to provide data in the appropriate form. When data were unavailable (e.g. standard deviations) estimations were calculated using recommended methods in the Cochrane Handbook (Higgins & Green, 2011). Included C-RCTs were assessed for unit of analysis error and adjusted as required.

Two pairs of independent authors assessed the risk of bias and overall quality of the evidence using the Cochrane Collaboration’s tool (KO and FT, RH) (Higgins & Green, 2011). A third reviewer resolved any disagreement (CW). We also considered sources of other bias, including whether the intervention was delivered as intended, whether groups were comparable at baseline and whether contamination between groups occurred.

The overall quality of the evidence for each pooled analysis was assessed using the GRADE criteria (Guyatt et al., 2011). The quality of evidence was downgraded by one level when appropriate according to the following criteria: study design limitations, inconsistency of results, imprecision, indirectness, and publication bias, resulting in the quality of evidence being judged as ‘high quality’, ‘moderate quality’, ‘low quality’, or ‘very low quality’. We used the *I*^2^ and Tau^2^ statistic to assess heterogeneity between trials, and Tau^2^ > 1 and *I*^2^ > 50% was used to identify high heterogeneity (Higgins et al., 2003). We planned to examine Egger’s test to assess publication bias if a sufficient number of studies (*n* ≥ 10) were included (Egger et al., 1997).

### Data synthesis and analysis

If studies reported data for multiple follow-up points, data from the longest time point was extracted for inclusion in meta-analyses (Kroon et al., 2014). Included studies were synthesised according to the intervention components (e.g. telephone or videoconferencing with educational materials or face-to-face contact) and by type of control: usual care (no treatment, usual care, wait-list control, or an attention control) or, other intervention (intervention with no telephone or videoconferencing component) (KO, CW, RH). To limit clinical heterogeneity we did not pool studies with clearly different intervention focus (e.g. physical activity vs. medication support).

Where different measures were used to measure the same outcome (i.e. disability), the most appropriate measure was selected based on the strength of their measurement properties (i.e. reliability, validity, and responsiveness) and its frequency of use in the included studies (to improve comparability across trials) (Fisher et al., 2014). For outcomes assessed using standard scales (e.g. Western Ontario and McMaster Universities Osteoarthritis Index), we used overall scores when possible. When measures presented did not point in the same direction (i.e. if some measures increase with disease severity whilst others decreased) we multiplied the mean values from one set of studies by −1 to ensure all were in the same direction (Higgins & Green, 2011). For the outcome psychological symptoms, if both anxiety and depression were presented separately, we chose to extract data on depression (Kroon et al., 2014).

Where possible, for each continuous outcome we calculated standardised mean differences (SMDs) (which allowed us to combine different measures of the same outcome) and 95% confidence intervals (CIs) and used the random effects model to pool estimates for each analysis using RevMan version 5.3.5. Generic inverse variance method was used to account for the inclusion of both C-RCTs and RCTs. In all instances where we could not combine data in a meta-analysis, including data from non-randomised trials, we provided a narrative summary of the trial findings according to the review objectives. We conducted primary meta-analyses combining all conditions with similar intervention and comparators. We also present meta-analyses by condition where possible (i.e. osteoarthritis and spinal pain separately). Cohen’s *d* was used to classify the intervention effect sizes as small (*d* = 0.2), medium (*d* = 0.5), and large (*d* ≥ 0.8) (Carson, 2012).

We planned to perform subgroup analyses to explore the potential effect of modality of intervention delivery (telephone and videoconferencing), intervention type (single (e.g. telephone only) and multicomponent (e.g. telephone-based intervention and educational materials)), and duration of spinal pain (acute and chronic (i.e. pain that lasted longer than 3 months)). Sensitivity analyses were planned to explore the influence of overall high risk of bias on pooled treatment effects. Overall risk of bias was defined as being at high risk of bias for one or more key domains (i.e. selection bias, performance bias, detection bias, attrition bias, reporting bias) (Higgins & Green, 2011). Sensitivity analyses were planned to explore the influence of small trials (sample size <100 per group) on pooled treatment effects, as it has been previously reported that small trials tend to report larger benefits of treatment than larger trials in osteoarthritis research (Nüesch et al., 2010).

## Results

**Figure 1:**
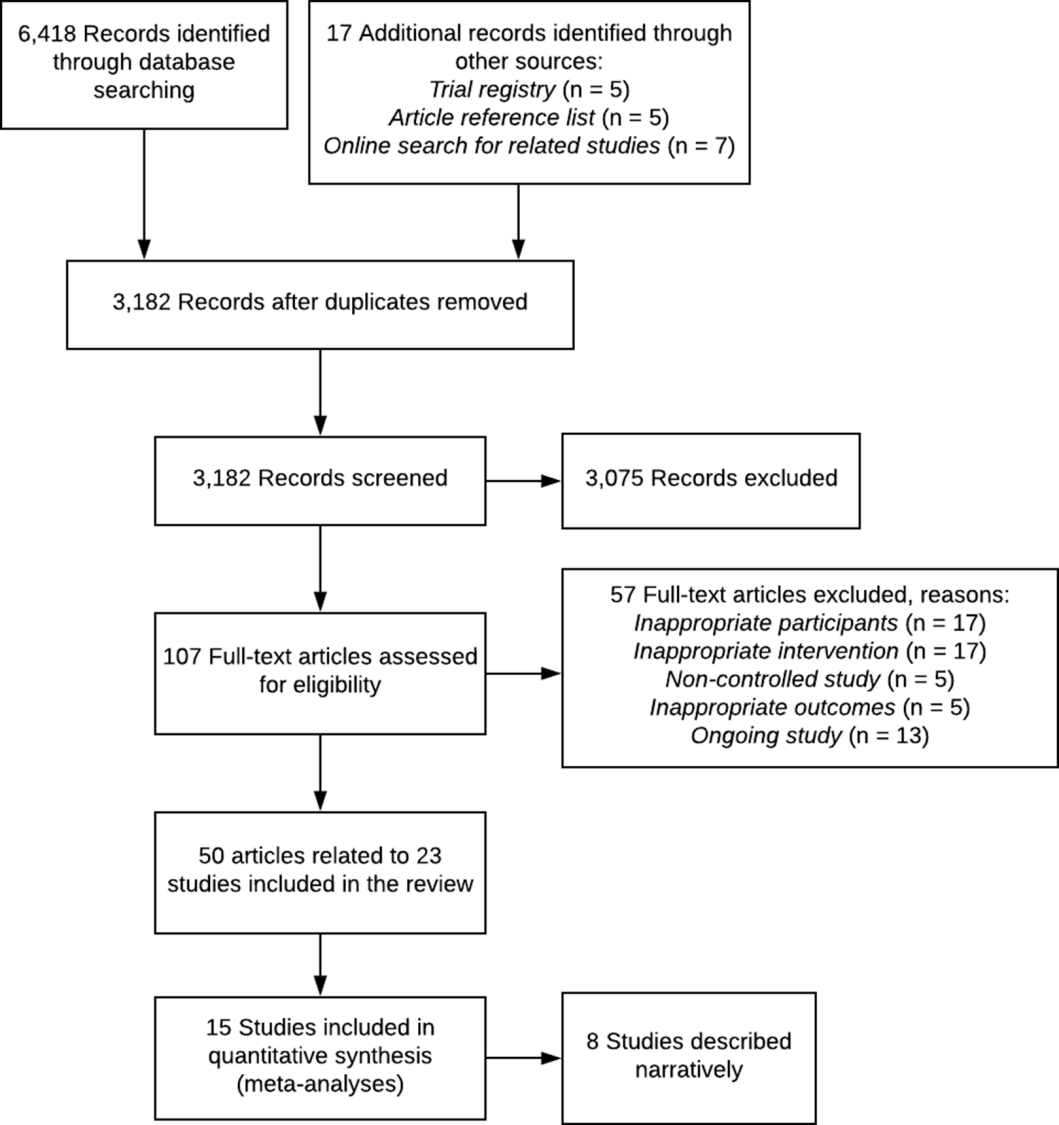
PRISMA flow diagram.

After duplicate removal, our search identified 3,182 records. Title and abstract screening excluded 3,075 records. Of the 107 full-text records assessed for eligibility, 57 were excluded (see Fig. 1). A total of 50 records (representing 23 studies) fulfilled the eligibility criteria and were included in the review (Fig. 1). Overall 20 RCTs, 2 C-RCT and 1 non-randomised controlled trial with a total of 4,994 participants (range 30–786) across 56 trial arms were included (Table 1). Trials were published between 1989 and 2018. A total of 12 studies were undertaken in the United States, four in Australia, and one each in Sweden, the Netherlands, Italy, Canada, Nigeria, Brazil, and England (Table 1). Intervention duration ranged from 4 weeks to 2 years, with most (*n* = 14) having a duration of 6 weeks to 6 months (Table 1). No studies experimentally compared interventions of varying number of calls. Five studies reported secondary analyses that examined associations of telephone call dose with patient outcomes (Thomas et al., 2002; Damush et al., 2003; Hughes et al., 2010; Williams et al., 2016; O’Brien et al., 2018). Three studies found no association between the number of calls and patient outcomes (Damush et al., 2003; O’Brien et al., 2018; Williams et al., 2018) and two studies indicated that completion of a higher number of calls was associated with greater improvements in pain (Thomas et al., 2002; Hughes et al., 2010), physical function (Hughes et al., 2010) and depression (Hughes et al., 2010).

**Table 1:** Characteristics of trials included the review.

Author (year) Design Country Total participants	Patient condition Diagnosis Mean age (years) %female Recruitment Study arms Participation rate (%)^a^	Intervention Content	Intervention Intensity/duration Delivery agent Other components	Comparison group(s)	Length of follow-up Attrition at final follow-up (%)^b^
Allen et al. (2010) RCT USA *n* = 523	Knee and/or hip OA Clinical assessment and radiographic evidence 60.1 7 Recruited from the Durham Veterans Affairs Medical Center 3 80	Information was grounded in SCT and focused on self-efficacy, managing osteoarthritis symptoms, goal setting and perceived facilitators and barriers. Modules included basic self-management, exercise, healthy eating and weight management, medications, joint injections and surgery, communication with health care providers, joint care and protection, complementary and alternative medicines, stress management and relaxation, sleep.	12 monthly calls over 12 months Health educator Written and audio education and an exercise video	Two comparison groups: 1. Usual care 2. Attention control: received written and audio education modules for common health problems and 12 monthly calls over 12 months to review education modules Both groups were combined for meta-analysis	12 months 2 VAS = 18
Allen et al. (2016) C-RCT USA *n* = 300	Knee and/or hip OA Clinical assessment and radiographic evidence 61.1 9 Recruited from the Durham Veterans Affairs Medical Center 2 84	Intervention focused on physical activity, weight management and cognitive behavioural pain management strategies, goal-setting and used MI strategies.	18 calls over 12 months (two calls per month for first 6 months, then monthly calls for last 6 months) Counsellor Written education, an exercise video, an audio CD of relaxation exercises	Usual care	12 months 9
Allen et al. (2017) C-RCT USA *n* = 537	Knee and/or hip OA Clinical assessment and radiographic evidence 63.3 74 Recruited from Duke University Health System community-based primary care clinics 4 NR; 82	Two telephone groups: 1. Patient intervention: Focused on physical activity, weight management, and cognitive behavioural strategies for managing pain. Goal setting and action planning were major intervention components. Motivational interviewing strategies were used throughout the intervention. 2. Patient-provider intervention: combination patient intervention and provider intervention (see comparison groups). Patient and patient-provider groups were combined for meta-analysis.	18 calls over 12 months (2 calls per month for first 6 months, then monthly calls for last 6 months) Counsellor Written education, an exercise video, an audio CD of relaxation exercises	Two comparison groups: 1. Provider intervention: providers received patient specific osteoarthritis treatment recommendations based on treatment guidelines however decisions regarding whether to recommend these treatments to patients were at the discretion of the providers 2. Usual care (comparison group for meta-analysis)	12 months 7
Bennell et al. (2017) RCT Australia *n* = 168^c^	Knee OA Based on clinical assessment 62.3 73 Recruited from advertisements in print, radio and social media, and trial research volunteer database 2 NR; 94	The physiotherapist provided education about osteoarthritis, benefits of physical activity/exercise and strategies to enhance adherence, prescribed an exercise program and assisted with goal setting and individual barriers.	6 monthly calls over 6 months Health professional Option of up to six additional calls, 5 x 30 min physiotherapy sessions including written education	Face-to-face Iv alone: 5 × 30 min physiotherapy sessions including written education	18 months 24
Blixen et al. (2004) RCT USA *n* = 32^c^	OA Physician assessment (diagnosis criteria not stated) 70.8 38 Recruited from arthritis/ rheumatology clinics in Midwestern hospitals 2 64	Self-management program including information on pathology, osteoarthritis medication, the interrelationship between emotional and physical components of pain, the importance of relaxation techniques, depression, the importance of regular exercise, weight management, goal setting, and communicating with health-care providers.	6 weekly calls over 6 weeks Advanced practice nurse Written and audio education	Usual care	6 months 3
Buhrman et al. (2004) RCT Sweden *n* = 56^c^	Chronic back pain Physician assessment (have back pain ≥3 months) 44.6 35 Recruited via newspapers and webpages 2 84	Based on CBT, the intervention included information about pain, physical exercise, activity pacing, ergonomics, external focusing and cognitive reconstruction, stress management, problem solving, sleeping disorders and maintaining coping strategies.	6 weekly calls over 6 weeks Graduate students trained in CBT Education via the internet and CD	Waiting-list control	2 months 9
Burks (2001) RCT USA *n* = 120^c^	Knee OA Based on clinical assessment 66.7 8 Recruited from Veteran Affairs primary care and specialty clinics 3 86	The self-management program included pain management, mobility and function, as well as tension and mood.	Three fortnightly calls over 6 weeks Primary care provider Written materials	Two comparison groups: 1. Usual care 2. Attention control: received written materials and 3 fortnightly calls over 6 weeks to answer questions only	3 months 12.5
Cuperus et al. (2015) RCT Netherlands *n* = 158^c^	OA Based on clinical assessment 60.0 90 Recruited from outpatient visits 2 67	The healthcare providers were trained in techniques of MI and teaching self-management principles. Session included information about osteoarthritis, pain management, medication, physical activity, activity pacing, food consumption, and goal setting regarding pain management and physical activity.	4 weekly calls over 4 weeks Rheumatology nurse 2 weekly in-clinic sessions over 2 weeks	Face-to-face Iv alone: received 6 weekly in-clinic sessions over 6 weeks	12 months 13
Damush et al. (2003) RCT USA *n* = 211	Acute low back pain Physician assessment (have back pain <3 months) 45.5 73 Recruited from the Regenstrief medical record system 2 52	The intervention based on a chronic back pain program and SCT focused on increasing self-efficacy and social support to self-manage low back pain. Further information on exercises, goal setting, problem solving and strategies to resolve potential barriers.	3 biweekly telephone calls (week 4, 6, 8) then 2 monthly calls over 2 months NR 3 weekly in-person classes over 3 weeks, written and audio materials	Usual care	12 months 34
Gialanella et al. (2017) RCT Italy *n* = 100^c^	Chronic neck pain Physician assessment (have back pain ≥6 months) 58.1 88 Recruited from physician consults 2 NR; could not be calculated	Education about regular physical activity and exercises and advice on solutions for persistent pain and any symptoms of exacerbation, use of medications.	12 fortnightly calls over 6 months Nurse Optional additional calls Written and illustrated material explaining exercises	Usual care	6 months 6
Goode et al. (2018) RCT USA *n* = 60^c^	Chronic low back pain Self-reported low back pain on most days for >3 months 70.3 7 Recruited from the Durham Veterans Affairs Medical Center 3 86	Two telephone groups: Physical activity group: intervention included a personalised exercise program, exercise and activity goal setting, assessment of exercise barriers, and education on condition, function, gate theory of pain and exercise and healthy eating. Physical activity + CBT group: intervention included the same as the physical activity group + CBT for pain skills e.g. overcoming pain-related barriers, progressive muscle relaxation.	Three calls every 4 weeks from physical therapist and 10 calls from exercise counsellor over 12 weeks Physical therapist and exercise counsellor One in-person session at baseline, written and illustrated materials explaining exercises, exercise video	Waiting-list control	3 months 17
Hughes et al. (2010) RCT USA *n* = 419	Knee and/or hip OA Based on clinical assessment 71.1 87 Recruited from local newspaper, media, and senior groups 2 86	Physical activity maintenance, facilitators/barriers to exercise. Training focused on background and application of the trans theoretical model and MI principles, described how to monitor physical activity participation, and provided strategies for setting goals, solving problems, and reinforcing progress.	Six fortnightly calls over 3 months and 12 monthly calls over 12 months Fit and Strong! Instructor Group 1 Negotiated with telephone Individualised plans for physical activity. Option to attend a facility-based class, use facility- or home-based equipment over 16 months Group 2 Mainstream with telephone Referred to an existing group/facility-based best-practice program offered at the same senior centre for 1hr 3×/week over 16 months	Face-to-face Iv alone: Group 1 Negotiated no telephone Individualised plans for physical activity. Option to attend a facility-based class, use facility- or home-based equipment over 16 months Group 2 Mainstream no telephone Referred to an existing group/facility-based best-practice program offered at the same senior centre for 1hr 3x/week over 16 months	18 months 38
Iles et al. (2011) RCT Australia *n* = 30^c^	Acute low back pain Physiotherapist assessment (onset back pain within previous 8 weeks) 39.5 40 Recruited from physiotherapy outpatient clinic 2 NR; 77	Coaching included techniques such as MI to increase the perceived importance of the activity and cognitive behavioural strategies to increase confidence in activity, and goal setting and potential barriers to return to activity.	4 weekly calls over 4 weeks (week 1–4) then one call at week 7 Physiotherapist No other components	Usual care	3 months 13
Li et al. (2017) RCT Canada *n* = 34^c^	Knee OA Physician confirmed or clinical assessment 55.5 82 Recruited from social media and emails from the arthritis and research groups 2 74	Intervention included education about physical activity, including the benefits of physical activity, the detrimental effects of sedentary behaviour, and ways to be active without aggravating OA symptoms. Calls focused on activity goals, identifying barriers and solutions, and building confidence to implement the physical activity plan.	4 weekly calls over 4 weeks Physiotherapy 1 in-clinic sessions at baseline + a Fitbit to wear to track physical activity behaviour	Waiting-list control	1 month 0
Maisiak, Austin & Heck, (1996) RCT USA *n* = 186^c^	Knee and/or hip OA Physician assessment (diagnosis criteria not stated) 60.5 92 Recruited from the Arthritis Information Service 3 ∼75	Intervention included 6 categories of patient behaviour: patient-physician communication, medication compliance, and removing barriers to medical care, symptom reviews, self-care activities, and stress control.	Five fortnightly calls over 3 months (first call at week 2), then six calls at 4-week intervals over 6 months Counsellor No other components	Two comparison groups: 1. Usual care 2. Attention control: received 11 contacts over 9 months for the purpose of symptom monitoring only Both groups were combined for meta-analysis	9 months 6
Mazzuca et al. (1997) Non-RCT USA *n* = 211	Knee OA Clinical assessment and radiographic evidence 62.5 85 Recruited from the Regenstrief medical record system 2 NR; could not be calculated	Core content areas included quadriceps-strengthening exercises, control of joint pain with thermal modalities, and joint protection, and medication use for those prescribed.	Two calls over 1 month (at week 1 and at 1 month) Arthritis nurse educator 1 in-clinic education session and pamphlet	Attention control: received an audio-visual presentation, a newsletter and a call at week 1 and at 1 month to reinforce participation only	12 months 19
O’Brien et al. (2018) RCT Australia *n* = 120^c^	Knee OA Physician assessment and self-reported pain due to knee OA >3 months 61.6 62 Recruited from outpatient orthopaedic consultation waiting list 2 88	Brief telephone advice and education about the benefits of weight loss and physical activity for knee osteoarthritis and referral to the NSW Get Healthy Service which aims to support adults to make sustained lifestyle improvements including diet, physical activity and achieving a healthy weight, and where appropriate, access to smoking cessation services	Brief call at baseline + 10 calls over 6 months Trained telephone interviewers (brief education) + University qualified health coaches Printed support material	Usual care	6 months 3
Odole & Ojo (2013) RCT Nigeria *n* = 50^c^	Knee OA Physician assessment (diagnosis criteria not stated) 55.5 48 Recruited from physiotherapy outpatient clinic 2 NR; could not be calculated	Standardised home-exercise program.	Three calls a week over 6 weeks Physiotherapist Standardised exercise program manual and exercise log book	Face-to-face Iv alone: received standardised exercise program in the clinic 3 times a week for 6 weeks	6 weeks 0
De Rezende et al. (2016) RCT Brazil *n* = 228^c^	Knee OA Clinical assessment and radiographic evidence 65 NR Recruited during clinical care and from telephone calls 2 75	Lectures and workshops on the anatomy of a joint and the pathology of osteoarthritis, its causes, irreversibility, and management, coping skills, medication, importance of physical activity, protecting joints, well-balanced diet, and how patients could and should include habits of regular leisure, sports and social gathering, and tasks.	Group 1a 1 call 2 months after final lecture Health professional 2 lectures 1 month apart, educational handout and video Group 2a 1 call 2 months after final lecture Health professional 2 lectures 2 months apart, educational handout and video Group 3a 1 call 2 months after final lecture Health professional 2 lectures 3 months apart, educational handout and video Group 4a 1 call 2 months after receiving intervention material Health professional Educational handout and video	Face-to-face Iv alone: Group 1b Received 2 lectures 1 month apart, educational handout and video Group 2b Received 2 lectures 2 months apart, educational handout and video Group 3b Received 2 lectures 3 months apart, educational handout and video Group 4b Received an educational handout and video	12 months 13
Rutledge et al. (2018) RCT USA *n* = 66^c^	Chronic low back pain Clinical assessment 53.3 38 Recruited by flyers posted in primary care clinic waiting areas, clinician referrals, and paid advertisements in public media 2 81%	The core content topics included pain management, stress management, cognitive changes, assertive communication, and goal setting.	11 calls over 8 weeks Mental health therapist 1 in-clinic session and written education materials	Attention control: Supportive Care treatment included education by distribution of a standard text, The Back Pain Help Book, active listening by the therapist to participant’s concerns, support, recommendations to follow the advice of their caretakers providing usual medical care	2 months 14
Thomas et al. (2002) RCT England *n* = 786	Knee OA Questions were used to define patients (“Have you ever had pain in or around the knee on most days for at least a month”) 62 64 Recruited by mail from general practice records 4 NR; 93	Exercise programme, advice on the management of knee pain.	24 monthly calls over 24 months Trained researcher Exercise program; four home visits in the first 2 months, plus follow-up visits at 6 monthly intervals	Face-to-face Iv alone: Included 2 groups; exercise only group: four home visits in the first 2 months, plus follow-up visits at 6 monthly intervals and control group: no intervention. Both groups were combined for analysis by authors	24 months 13
Weinberger et al. (1989) RCT USA *n* = 439	OA Clinical assessment and radiographic evidence 62.3 88 Recruited by primary care physicians 4 75	Two telephone groups: the telephone group and the telephone + clinic group. Both consisted of brief interviewers focusing on: medications (i.e. side effects, compliance, whether the supply was sufficient to last until the next appointment), joint pain, gastrointestinal symptoms, other chronic diseases, all scheduled outpatient visits, an existing process by which patients could telephone a GMP provider, barriers to keeping their clinic appointments.	11 monthly calls over 11 months Trained nonmedical personnel Scheduled in-clinic visits	Two comparison groups: 1. Usual care 2. Face-to-face Iv alone: attended scheduled in-clinic visits	11 months 10.3
Williams et al. (2018) RCT Australia *n* = 160^c^	Chronic low back pain Physician assessment and self-reported chronic low back pain >3 months 56.7 59 Recruited from outpatient orthopaedic consultation waiting list 2 89	Brief telephone advice including information that a broad range of factors contribute to the experience of low back pain and potential benefits of weight loss and physical activity for reducing low back pain and referral to the NSW Get Healthy Service which aims to support adults to make sustained lifestyle improvements including diet, physical activity and achieving a healthy weight, and where appropriate, access to smoking cessation services	Brief call at baseline + 10 calls over 6 months Trained telephone interviewers (brief education) + University qualified health coaches Clinical in-person consultation (Physiotherapist) and printed support material	Usual care	6 months 12

**Notes:**

CBT, cognitive behaviour therapy; C-RCT, cluster randomised controlled trial; GMP, General Medicine Practice; Iv, intervention; MI, motivational interviewing; NR, not reported; OA, osteoarthritis; RCT, randomised controlled trial; SCT, social cognitive theory; VAS, visual analogue scale.

a If not reported, participation rate was calculated as percentage participating of those reached and eligible.

b Attrition reported at each time-point for all outcomes or by individual outcomes if different.

c Small trial (sample size <100 per group).

Eight trials included patients with knee osteoarthritis (*n* = 1,717), five trials included patients with hip and/or knee osteoarthritis (*n* = 1,965), three did not specify osteoarthritis type (*n* = 629), two included patients with acute back pain (*n* = 241), four included patients with chronic back pain (*n* = 342), and one included patients with chronic neck pain (*n* = 100) (Table 1).

All 23 trials utilised telephone for intervention delivery (i.e. no studies utilised videoconferencing). A total of 16 trials compared a telephone-based intervention with usual care, six trials compared a telephone-based intervention to a face-to-face intervention, and one trial used a three-arm design comparing a telephone-based intervention to usual care and to a face-to-face intervention (Table 1). Only two trials tested telephone alone as the mode of intervention delivery, five tested telephone combined with face-to-face, nine tested telephone combined with educational materials and seven tested telephone combined with face-to-face and educational materials (Table 1). All studies, except one (O’Brien et al., 2018), implemented an intervention designed specifically for osteoarthritis and spinal pain patients. All interventions focused on supporting self-management and providing education in addition to a range of intervention targets, for example physical activity (see Table 1 for details). A total of 17 studies assessed pain intensity (Weinberger et al., 1989; Mazzuca et al., 1997; Thomas et al., 2002; Buhrman et al., 2004; Hughes et al., 2010; Allen et al., 2010, 2016, 2017; Odole & Ojo, 2013; Cuperus et al., 2015; De Rezende et al., 2016; Bennell et al., 2017; Gialanella et al., 2017; Li et al., 2017; O’Brien et al., 2018; Rutledge et al., 2018; Williams et al., 2018) and 21 studies assessed a disability outcome (Weinberger et al., 1989; Maisiak, Austin & Heck, 1996; Mazzuca et al., 1997; Burks, 2001; Damush et al., 2003; Blixen et al., 2004; Hughes et al., 2010; Allen et al., 2010, 2016, 2017; Iles et al., 2011; Odole & Ojo, 2013; Cuperus et al., 2015; De Rezende et al., 2016; Bennell et al., 2017; Gialanella et al., 2017; Li et al., 2017; O’Brien et al., 2018; Rutledge et al., 2018; Goode et al., 2018; Williams et al., 2018).

The majority of studies were rated high risk for performance bias (*n* = 22) and detection bias (*n* = 21) due to the inability to blind treatments and self-reported outcomes (Fig. S1). Two studies were rated low risk for detection bias as the intervention was part of a cohort multiple RCT and the participants were unaware of reciprocal study groups (O’Brien et al., 2018; Williams et al., 2018) and one was also rated low risk for performance bias as the personnel delivering the intervention were also unaware of the reciprocal study groups (O’Brien et al., 2018). The majority of studies were rated low for random sequence (*n* = 18), attrition bias (*n* = 17), and other bias (*n* = 16) and unclear for allocation concealment (*n* = 11) and reporting bias (*n* = 13). Egger’s test was not undertaken to assess publication bias as there was not a sufficient number of studies for any of the comparisons (*n* < 10) (Egger et al., 1997). Visual inspection of funnel plot asymmetry was not undertaken as there was not a sufficient number of studies to judge asymmetry (*n* < 10) (Higgins & Green, 2011). *I*^2^ and Tau^2^ statistics suggested statistical heterogeneity in the telephone-based interventions (with educational materials) vs. usual care comparison for pain intensity (*I*^2^ = 74%) and disability (*I*^2^ = 56%).

### Primary outcomes

#### Telephone-based interventions (with educational materials) vs. usual care

##### Pain intensity

Meta-analysis of data from five studies (Buhrman et al., 2004; Allen et al., 2010, 2016, 2017; Gialanella et al., 2017) (*n* = 3 knee and/or hip osteoarthritis, *n* = 2 spinal pain; total *n* = 1,357 patients) revealed a small positive intervention effect of telephone-based interventions (with educational materials) on pain intensity compared to usual care (SMD −0.27, 95% CI [−0.53, −0.01], Tau^2^ = 0.06, *I*^2^ = 74%; moderate-quality evidence) (Table 2; Fig. S2). Positive intervention effects were found for spinal pain (SMD −0.55, 95% CI [−0.92, −0.19]) but not osteoarthritis when synthesised separately (Table 2; Fig. S2). All planned subgroup analyses were not possible due to the limited number of included studies (Table 2). To reduce risk of clinical heterogeneity, one study (O’Brien et al., 2018) which was not disease specific was not included in the main meta-analyses. This study reported no difference in pain intensity for the telephone-based intervention compared to usual care (Table S2).

**Table 2:** Summary of meta-analysis finding.

Overall/subgroup/sensitivity analyses^a^	No. of patients (trials)	Effect sizes [95% CI]	Quality of the evidence GRADE
**Telephone-based interventions (with educational materials) vs. usual care**			
**Pain (primary outcome)**			
Overall analysis	1,357 (5 trials)	−0.27 [−0.53, −0.01]^b^	⊕⊕⊕⊖ Moderate^c^
Patient condition			
Osteoarthritis	1,212 (3 trials)	−0.16 [−0.47, 0.14]	
Spinal pain	145 (2 trials)	−0.55 [−0.92, −0.19]^b^	
Sensitivity analyses			
Excluding small trials	1,212 (3 trials)	−0.16 [−0.47, 0.14]	
**Disability (primary outcome)**			
Overall analysis	1,537 (7 trials)	−0.21 [−0.40, −0.02]^b^	⊕⊕⊕⊖ Moderate^c^
Patient condition			
Osteoarthritis	1,417 (5 trials)	−0.13 [−0.30, 0.04]	
Spinal pain	120 (2 trials)	−0.64 [−1.01, −0.27]^b^	
Subgroup analyses			
Intervention type			
Single component	201 (2 trials)	−0.30 [−0.59, −0.01]^b^	
Multicomponent	1,492 (5 trials)	−0.18 [−0.42, 0.06]	
Sensitivity analyses			
Excluding small trials	1,212 (3 trials)	−0.10 [−0.34, 0.14]	
**Psychological symptoms (secondary outcome)**			
Overall analysis	1,293 (5 trials)	0.03 [−0.10, 0.16]	⊕⊕⊕⊖ Moderate^d^
Patient condition			
Osteoarthritis	1,242 (4 trials)	0.03 [−0.13, 0.19]	
Sensitivity analyses			
Excluding small trials	1,212 (3 trials)	0.02 [−0.16, 0.20]	
**Self-efficacy (secondary outcome)**			
Overall analysis	571 (3 trials)	0.20 [0.03, 0.38]^b^	⊕⊕⊕⊕ High
Patient condition			
Osteoarthritis	545 (2 trials)	0.19 [0.01, 0.36]^b^	
Subgroup analysis			
Intervention type			
Multicomponent	545 (2 trials)	0.19 [0.01, 0.36]^b^	
**Weight loss (secondary outcome)**			
Overall analysis	697 (2 trials)	−0.07 [−0.25, 0.11]	⊕⊕⊕⊖ Moderate^d^
**Telephone plus face-to-face interventions vs. usual care**			
**Pain (primary outcome)**			
Overall analysis	259 (3 trials)	−0.08 [−0.32, 0.16]	⊕⊕⊕⊖ Moderate^d^
Patient condition			
Spinal pain	225 (2 trials)	−0.09 [−0.36, 0.17]	
**Disability (primary outcome)**			
Overall analysis	398 (4 trials)	−0.08 [−0.28, 0.12]	⊕⊕⊕⊖ Moderate^d^
Patient condition			
Spinal pain	364 (3 trials)	−0.11 [−0.31, 0.10]	
Subgroup analyses			
Spinal pain duration (chronic)	225 (2 trials)	0.00 [−0.26, 0.26]	
**Psychological symptoms (secondary outcome)**			
Overall analysis	298 (2 trials)	−0.12 [−0.35, 0.11]	⊕⊕⊕⊖ Moderate^d^
**Telephone plus comprehensive face-to-face interventions vs. face-to-face interventions alone**			
**Pain (primary outcome)**			
Overall analysis^e^	513 (3 trials)	−0.13 [−0.30, 0.04]	⊕⊕⊕⊖ Moderate^d^
**Disability (primary outcome)**			
Overall analysis^e^	513 (3 trials)	−0.06 [−0.31, 0.19]	⊕⊕⊕⊖ Moderate^d^
**Psychological symptoms (secondary outcome)**			
Overall analysis^e^	345 (2 trials)	0.11 [−0.10, 0.32]	⊕⊕⊕⊖ Moderate^d^

**Notes:**

a Planned subgroup and sensitivity analyses were not conducted due to insufficient study numbers: telephone-based interventions (with educational materials) vs. usual care (*n* = 38); patient condition (spinal pain (*n* = 3)), by intervention type (single (*n* = 4) and multicomponent (*n* = 4)), by modality (telephone (*n* = 5) and videoconferencing (*n* = 5)), by condition duration (acute (*n* = 5) and chronic (*n* = 5)), by high risk of bias (*n* = 5), and by trial size (*n* = 2). Telephone plus face-to-face interventions vs. usual care (*n* = 28): patient condition (osteoarthritis (*n* = 3), spinal pain (*n* = 1)), by intervention type (single (*n* = 3) and multicomponent (*n* = 3)), by modality (telephone (*n* = 3) and videoconferencing (*n* = 3)), by condition duration (acute (*n* = 3) and chronic (*n* = 2)), by high risk of bias (*n* = 4), and by trial size (*n* = 3). Telephone plus comprehensive face-to-face interventions vs. face-to-face interventions alone (*n* = 24); by patient condition (osteoarthritis (*n* = 3), spinal pain (*n* = 3)), by intervention type (singular (*n* = 3) and multicomponent (*n* = 3)), by modality (telephone (*n* = 3) and videoconferencing (*n* = 3)), by high risk of bias (*n* = 3) and by trial size (*n* = 3).

b Significant at *p* < 0.05.

c Downgraded due to inconsistency of results: *I*^2^ > 50%.

d Downgraded due to imprecision: the confidence intervals contained the null value.

e One study (De Rezende et al., 2016) which compared two interventions entered into RevMan.

##### Disability

Meta-analysis of data from seven studies (Maisiak, Austin & Heck, 1996; Blixen et al., 2004; Allen et al., 2010, 2016, 2017; Iles et al., 2011; Gialanella et al., 2017) (*n* = 4 trials knee and/or hip osteoarthritis, *n* = 1 trial unspecified osteoarthritis, *n* = 2 trials spinal pain; total *n* = 1,537 patients) found a small positive intervention effect of telephone-based interventions (with educational materials) compared to usual care on disability (SMD −0.21, 95% CI [−0.40, −0.02], Tau^2^ = 0.03, *I*^2^ = 56%; moderate-quality evidence) (Table 2; Fig. S2). Positive intervention effects were found for spinal pain (SMD −0.64, 95% CI [−1.01, −0.27]) but not osteoarthritis when synthesised separately (Table 2; Fig. S2). Subgroup analyses revealed a positive intervention effect for single component interventions (SMD −0.30, 95% CI [−0.59, −0.01]) and no difference between multicomponent interventions compared to usual care (Table 2; Fig. S3). Two studies (Burks, 2001; O’Brien et al., 2018) were not included in main meta-analyses due to insufficient data reporting (e.g. only reported *p*-values) or not disease specific. Both of these studies reported no difference in disability between telephone-based interventions and usual care (Table S2).

#### Telephone plus face-to-face interventions vs. usual care

##### Pain intensity

Meta-analysis of data from three studies (Li et al., 2017; Rutledge et al., 2018; Williams et al., 2018) (*n* = 1 trial knee osteoarthritis, *n* = 2 trials spinal pain; total *n* = 259 patients) showed no difference between telephone plus face-to-face interventions on pain intensity compared to usual care (SMD −0.08, 95%CI [−0.32, 0.16], Tau^2^ = 0.00, *I*^2^ = 0%; moderate-quality evidence) (Table 2; Fig. S4). Similar to the main analysis, no intervention effect was found for spinal pain (Table 2; Fig. S4). All planned subgroup analyses were not possible due to the limited number of included studies (Table 2). Three studies (Weinberger et al., 1989; Mazzuca et al., 1997; Thomas et al., 2002) were not included in main meta-analyses due to non-randomised design or did not report sufficient data (e.g. means at follow-up not reported). All three studies reported no difference in pain intensity between the telephone plus face-to-face interventions compared to usual care (Table S2).

##### Disability

Meta-analysis of data from four studies (Damush et al., 2003; Li et al., 2017; Rutledge et al., 2018; Williams et al., 2018) (*n* = 1 trial knee osteoarthritis, *n* = 3 trials spinal pain; total *n* = 398 patients) showed no difference between telephone plus face-to-face interventions compared to usual care on disability (SMD −0.08, 95%CI [−0.28, 0.12], Tau^2^ = 0.00, *I*^2^ = 0%; moderate-quality evidence) (Table 2; Fig. S4). Similar to the main analysis, no intervention effect was found for spinal (Table 2; Fig. S4). Subgroup analyses also revealed no intervention effects for interventions including chronic spinal pain patients (Table 2; Fig. S5). Four studies (Weinberger et al., 1989; Mazzuca et al., 1997; Thomas et al., 2002; Goode et al., 2018) were not included in main meta-analyses due to non-randomised design or did not report sufficient data (e.g. means at follow-up not reported) (Table S2). Three studies reported no difference in disability between the telephone plus face-to-face interventions compared to usual care (Weinberger et al., 1989; Mazzuca et al., 1997; Thomas et al., 2002). One study reported telephone plus face-to-face interventions significantly improved disability compared to usual care (Goode et al., 2018) (Table S2).

#### Telephone plus comprehensive face-to-face interventions vs. face-to-face interventions alone

##### Pain intensity

Meta-analysis of data from three studies (Cuperus et al., 2015; De Rezende et al., 2016; Bennell et al., 2017) (*n* = 2 trials knee osteoarthritis, *n* = 1 trial unspecified osteoarthritis; total *n* = 513 patients) showed no difference between telephone plus comprehensive face-to-face interventions and face-to-face interventions alone to reduce pain intensity (SMD −0.13, 95% CI [−0.30, 0.04], Tau^2^ = 0.00, *I*^2^ = 0%; moderate-quality evidence) (Table 2; Fig. S6). No subgroup analyses were possible for any comparative effectiveness comparisons (Table 2). A further two studies (Hughes et al., 2010; Odole & Ojo, 2013) were not included in the meta-analysis due to dissimilar intervention components or did not report sufficient data (e.g. means at follow-up not reported). Both reported no difference in pain intensity between telephone plus comprehensive face-to-face interventions and face-to-face interventions alone (Table S2).

##### Disability

Meta-analysis of data from three studies (Cuperus et al., 2015; De Rezende et al., 2016; Bennell et al., 2017) (*n* = 2 trials knee osteoarthritis, *n* = 1 trial unspecified osteoarthritis; total *n* = 513 patients) showed no difference between telephone plus comprehensive face-to-face interventions and face-to-face interventions alone to reduce disability (SMD −0.06, 95% CI [−0.31, 0.19], Tau^2^ = 0.04, *I*^2^ = 46%; moderate-quality evidence) (Table 2; Fig. S6). A further two studies (Hughes et al., 2010; Odole & Ojo, 2013) were not included in the meta-analysis due to dissimilar intervention components or did not report sufficient data (e.g. means at follow-up not reported). Both reported no difference in disability between telephone-based interventions and face-to-face interventions (Table S2).

### Secondary outcomes

#### Telephone-based interventions (with educational materials) vs. usual care

Meta-analysis of data from five studies (Blixen et al., 2004; Buhrman et al., 2004; Allen et al., 2010, 2016, 2017) (*n* = 3 trials hip and/or knee osteoarthritis, *n* = 1 trial unspecified osteoarthritis, *n* = 1 trials spinal pain; total *n* = 1,293 patients) showed no difference between telephone-based interventions (with educational materials) and usual care in improving psychological symptoms (SMD 0.03, 95% CI [−0.10, 0.16], Tau^2^ = 0.00, *I*^2^ = 4%; moderate-quality evidence) (Table 2; Fig. S2). Similar to the main analysis, results by patient condition showed no difference between telephone-based interventions and usual care in improving psychological symptoms for osteoarthritis (Table 2; Fig. S2). All planned subgroup analyses were not possible due to the limited number of included studies (Table 2).

Meta-analysis of three studies (Blixen et al., 2004; Allen et al., 2010; Iles et al., 2011) (*n* = 1 trial knee and/or hip osteoarthritis, *n* = 1 trial unspecified osteoarthritis, *n* = 1 trial spinal pain; total *n* = 571 patients) found a small positive effect of telephone-based interventions (with educational materials) on self-efficacy compared to usual care (SMD 0.20, 95% CI [0.03, 0.38], Tau^2^ = 0, *I*^2^ = 0%; high-quality evidence) (Table 2; Fig. S2). Similar to the main analysis, positive intervention effects were found for osteoarthritis (Table 2; Fig. S2). Subgroup analyses also revealed positive intervention effects for multicomponent interventions (Table 2; Fig. S7).

Meta-analysis of data from two studies (Allen et al., 2016, 2017) (*n* = 2 trials hip and/or knee osteoarthritis; total *n* = 697 patients) showed no difference between telephone-based interventions (with educational materials) and usual care in weight loss (SMD −0.07, 95% CI [−0.25, 0.11], Tau^2^ = 0, *I*^2^ = 0%; moderate-quality evidence) (Table 2; Fig. S2). All planned subgroup analyses were not possible due to the limited number of included studies (Table 2).

Four other studies not included in meta-analysis (reasons included not reporting a measure of variance at follow-up and not disease specific), reported no difference between groups for psychological symptoms (Maisiak, Austin & Heck, 1996), weight loss (O’Brien et al., 2018), physical activity (Allen et al., 2017; O’Brien et al., 2018), healthcare utilisation (Allen et al., 2016, 2017; O’Brien et al., 2018), or subjective improvement (O’Brien et al., 2018) (Table S2). A further three studies reported telephone-based interventions significantly improved psychological symptoms (O’Brien et al., 2018), physical activity (Allen et al., 2016), and recovery (Iles et al., 2011) compared to usual care (Table S2). One study also reported higher fear avoidance in the telephone-based intervention group compared to usual care (O’Brien et al., 2018). Two studies reported data on adverse events (Allen et al., 2016; O’Brien et al., 2018), one stated adverse events were similar between groups (O’Brien et al., 2018) and the other reported none of the adverse events were associated with the intervention (Allen et al., 2016). Three other studies stated no study-related adverse events occurred but did not provide any data (Allen et al., 2010, 2017; Gialanella et al., 2017) (Table S2).

#### Telephone plus face-to-face interventions vs. usual care

Meta-analysis of data from two studies (Damush et al., 2003; Williams et al., 2018) (*n* = 2 trials spinal pain; total *n* = 298 patients) showed no difference between telephone plus face-to-face interventions compared to usual care in improving psychological symptoms (SMD −0.12, 95% CI [−0.35, 0.11], Tau^2^ = 0, *I*^2^ = 0%; moderate-quality evidence) (Table 2; Fig. S4). All planned subgroup analyses were not possible due to the limited number of included studies (Table 2).

Five other studies not included in meta-analysis (reasons included not reporting a measure of variance at follow-up, non-randomised study design and dissimilar intervention components), reported no difference between groups for psychological symptoms (Weinberger et al., 1989), weight loss (Williams et al., 2018), physical activity (Li et al., 2017; Williams et al., 2018), healthcare utilisation (Williams et al., 2018), health-related quality of life (Mazzuca et al., 1997), subjective improvement (Rutledge et al., 2018; Williams et al., 2018), and fear avoidance (Williams et al., 2018) (Table S2). One study also reported telephone plus face-to-face interventions significantly improved physical activity and fear avoidance compared to usual care (Damush et al., 2003) (Table S2).

#### Telephone plus comprehensive face-to-face interventions vs. face-to-face interventions alone

Meta-analysis of data from two studies (Cuperus et al., 2015; De Rezende et al., 2016) (*n* = 1 trial knee osteoarthritis, *n* = 1 trial unspecified osteoarthritis; total *n* = 345 patients) showed no difference between telephone plus comprehensive face-to-face interventions and face-to-face interventions alone for psychological symptoms (SMD 0.11, 95% CI [−0.10, 0.32], Tau^2^ = 0, *I*^2^ = 0%, moderate-quality evidence) (Table 2; Fig. S6).

Other studies not included in the meta-analysis (reasons included insufficient data report, dissimilar intervention components, and not reporting a measure of variance) showed no difference between groups for psychological symptoms (Hughes et al., 2010; Odole & Ojo, 2013), self-efficacy (Cuperus et al., 2015), weight loss (Hughes et al., 2010; De Rezende et al., 2016), physical activity (Hughes et al., 2010; Cuperus et al., 2015; Bennell et al., 2017), healthcare utilisation (Bennell et al., 2017), health-related quality of life (Bennell et al., 2017), treatment adherence (Cuperus et al., 2015; De Rezende et al., 2016; Bennell et al., 2017), and fear avoidance (Cuperus et al., 2015) (Table S2). One study reported telephone-based interventions significantly improved global rating of change overall (Bennell et al., 2017) (Table S2). Of the four studies that reported adverse events, one study stated approximately one-third of participants reported mild adverse events (mostly transient increased knee pain) across both study groups during the intervention period (Bennell et al., 2017) and the remaining three studies described adverse events narratively with no data reported (Hughes et al., 2010; Cuperus et al., 2015; De Rezende et al., 2016) (Table S2).

#### Sensitivity analyses

We were unable to explore the influence of overall high risk of bias on pooled treatment effects as all studies were rated as high risk of bias (rated high risk of bias for one or more key domains). Sensitivity analyses to explore the impact of study size on our treatment effects are presented in Table 2. When excluding small trials, there was no longer an intervention effect of telephone-based interventions on pain intensity (Fig. S8), or disability (Fig. S3) compared to usual care. For psychological symptoms intervention effect was not changed by the removal of studies with small sample size (<100) (Fig. S9).

## Discussion

### Principle results

The trials in this review all used telephone-based interventions as the primary delivery method. All intervention content focused on self-management principles and providing education. Many studies included additional components such as educational materials and/or face-to-face interactions (21 of 23). We found moderate-quality evidence, as assessed using GRADE criteria (Guyatt et al., 2011), that telephone-based interventions (with educational materials) improves pain intensity, disability, and weight loss but not psychological symptoms compared to usual care. We also found high-quality evidence (Guyatt et al., 2011) that telephone-based interventions (with educational materials) improve self-efficacy compared to usual care. There was moderate-quality evidence (Guyatt et al., 2011) that there is no difference between telephone plus face-to-face interventions and usual care or face-to-face interventions alone in improving pain intensity, disability or psychological symptoms. There were limited studies that assessed the effects of telephone interventions on physical activity, health care or medication use or supporting treatment adherence.

Two previous systematic reviews of telehealth care for musculoskeletal conditions have been published (Cottrell et al., 2017a; Dario et al., 2017). A total of 10 of the studies on osteoarthritis and five on spinal pain were not included in these previous reviews. Cottrell et al. (2017a) found positive effects for real-time telephone or videoconferencing delivery of rehabilitation, mostly post-surgical (arthroplasty) rehabilitation for patients with various musculoskeletal conditions including back pain, neck pain, rheumatoid arthritis, and osteoarthritis. Our review considered a more homogenous patient group, the two most burdensome musculoskeletal conditions (osteoarthritis and spinal pain) and we excluded post-surgical rehabilitation. Our results, however, conflict with Dario et al. (2017) who included studies of any telehealth intervention for patients with non-specific low back pain and found no difference between these interventions and minimalist interventions (i.e. health or non-health-related information). The difference in results might be explained by our definition of a telehealth intervention, which was required to involve verbal telecommunication with direct patient-provider contact (i.e. telephone or videoconferencing), whereas Dario et al’s. definition of telehealth interventions included asynchronous methods such as email, e-community or web-based content. It is possible that the verbal person-to-person support via telephone explained the positive effects found in our review.

Our results show that telephone-based models of care improve patients outcomes, compared to usual care. Other evidence suggests telephone-based models of care are preferred by patients with chronic musculoskeletal conditions (Williams et al., 2014; Cottrell et al., 2017b). Despite this, telephone-based services are not widely available for patients with osteoarthritis or spinal pain. For other chronic conditions and health behaviours, such as diet and physical activity, telephone-based models have received significant investment based on additional evidence, including economic feasibility (Graves et al., 2009). While our review suggests that telephone-based interventions for people with osteoarthritis or spinal pain warrants consideration, there are unknown factors, for example, what the intervention should focus on, for example, weight or physical activity, that could impact the success of this in clinical practice. While our meta-analysis findings regarding self-efficacy point towards a possible mechanism in telephone-based interventions supporting capacity for self-management through self-efficacy, this and other possible mechanisms need to be tested formally.

The telephone is often used in clinical practice as an adjunct to face-to-face care (McLean et al., 2013). This is thought to allow more efficient integration of multidisciplinary roles and complex aspects of care (McLean et al., 2013; Dinesen et al., 2016). For osteoarthritis and spinal pain, our review suggests that for patients who already receive comprehensive face-to-face care additional telephone-based support does not improve pain and disability. We also found that telephone and face-to-face care was not better than usual care. However, we contend that the interventions in this meta-analysis are not comprehensive disease specific models, relative to other interventions we included. Overall, our findings do not support the additional use of telephone to clinical care that provides comprehensive management.

Usual care for many patients with osteoarthritis and spinal pain does not typically align with recommendations in clinical practice guidelines (Williams et al., 2010; Hunter, 2011; Brand et al., 2014). In light of the global disability burden of osteoarthritis and spinal pain, developing effective ways to deliver good quality care to the many patients with these conditions is an important future direction. Our results show that telephone services may be one way to provide remotely delivered care to people who cannot access it, or those who may otherwise receive suboptimal usual care. However, as usual care is often not evidence-based, arguably it may be an inferior control comparison to inform health policy and implementation (Williams et al., 2010; Hunter, 2011; Brand et al., 2014). One important research direction that would inform whether telephone-based models should be supported widely for musculoskeletal conditions is to understand if telephone only models are equivalent to good quality face-to-face care. We identified only one study assessing this comparison (Odole & Ojo, 2013). While the results of the study suggest equivalent effectiveness, more trials are needed to validate this finding, which also uses appropriate research designs (i.e. inferiority designs).

### Limitations

The majority of studies included in the meta-analysis focused on patients with osteoarthritis (16 of 23), so caution needs to be taken when generalising overall results to the management of patients with spinal pain. However, when reporting the findings separately for the two trials of spinal pain, the intervention effects for pain intensity and disability remained the same. All included studies were telephone-based interventions so we were unable to evaluate the effect of other verbal real-time telehealth interventions (i.e. videoconferencing interventions) as planned. Another limitation is inconsistent outcome reporting across the included studies. Around a third of included studies reported data that could not be synthesised in the meta-analysis for the core outcomes recommended to be used in clinical trials (e.g. OMERACT-OARSI, IMMPACT) (Dworkin et al., 2005); pain intensity (4/15), disability (5/19), psychological symptoms (3/12), and self-efficacy (1/3). Furthermore, only two included studies reported they collected data to assess cost-effectiveness. Future trials examining the effect of telephone-based interventions should undertake cost-effectiveness analyses to determine whether such interventions can improve patients’ outcomes at lower costs than usual care. Finally, in terms of GRADE, the overall quality of evidence was assessed as moderate for all meta-analyses but one (self-efficacy; high-quality of evidence). Although the effect we estimated is likely to be robust, there is a possibility that future high-quality research may change the effect estimates.

## Conclusions

Our review is the first to comprehensively synthesise evidence on telephone-based interventions for osteoarthritis and spinal, which are the most common musculoskeletal conditions and the leading causes of disability worldwide. There is moderate-quality evidence that telephone-based interventions, compared to usual care, are effective for pain and disability for osteoarthritis and spinal patients collectively. Telephone-based services offer the potential to support osteoarthritis and spinal pain patients to access better quality care.

## Supplemental Information

10.7717/peerj.5846/supp-1 Supplemental Information 1 PRISMA checklist.

10.7717/peerj.5846/supp-2 Supplemental Information 2 Search Strategy. Database(s): MEDLINE 1946 to Present with Daily Update.

10.7717/peerj.5846/supp-3 Supplemental Information 3 Any outcomes from a trial that could not be synthesised in meta-analysis.

10.7717/peerj.5846/supp-4 Supplemental Information 4 Risk of bias summary showing review authors’ judgments about each risk of bias domain in trials included the review. Trials are listed alphabetically by author name*. Notes: Green, low risk; yellow, unclear risk; red, high risk.

10.7717/peerj.5846/supp-5 Supplemental Information 5 Forest plots of main meta-analyses findings for comparison telephone-based interventions (with educational materials) versus usual care.

10.7717/peerj.5846/supp-6 Supplemental Information 6 Forest plots of disability outcome subgroup and sensitivity analyses for comparison telephone-based interventions (with educational material) versus usual care.

10.7717/peerj.5846/supp-7 Supplemental Information 7 Forest plots of main meta-analyses findings for comparison telephone plus face-to-face interventions versus usual care.

10.7717/peerj.5846/supp-8 Supplemental Information 8 Forest plots of disability outcome subgroup analysis comparison telephone plus face-to-face interventions versus usual care.

10.7717/peerj.5846/supp-9 Supplemental Information 9 Forest plots of main meta-analyses findings for comparison telephone plus comprehensive face-to-face interventions versus face-to-face interventions alone.

10.7717/peerj.5846/supp-10 Supplemental Information 10 Forest plots of self-efficacy outcome subgroup analysis for comparison telephone-based interventions (with educational material) versus usual care.

10.7717/peerj.5846/supp-11 Supplemental Information 11 Forest plots of pain intensity outcome sensitivity analysis for comparison telephone-based interventions (with educational material) versus usual care.

10.7717/peerj.5846/supp-12 Supplemental Information 12 Forest plots of psychological symptoms outcome sensitivity analyses for comparison telephone-based interventions (with educational material) versus usual care.

10.7717/peerj.5846/supp-13 Supplemental Information 13 Raw Data for systematic review.

## Additional Information and Declarations

### Data Availability

The following information was supplied regarding data availability: Raw data are provided in the Supplemental Files.

## References

[ref-1] Ackerman IN, Buchbinder R, Osborne RH (2013). Factors limiting participation in arthritis self-management programmes: an exploration of barriers and patient preferences within a randomized controlled trial. Rheumatology.

[ref-2] Allen KD, Oddone EZ, Coffman CJ, Datta SK, Juntilla KA, Lindquist JH, Walker TA, Weinberger M, Bosworth HB (2010). Telephone-based self-management of osteoarthritis: a randomized trial. Annals of Internal Medicine.

[ref-3] Allen KD, Oddone EZ, Coffman CJ, Jeffreys AS, Bosworth HB, Chatterjee R, McDuffie J, Strauss JL, Yancy WS, Datta SK, Corsino L, Dolor RJ (2017). Patient, provider, and combined interventions for managing osteoarthritis in primary care: a cluster randomized trial. Annals of Internal Medicine.

[ref-4] Allen KD, Yancy WS, Bosworth HB, Coffman CJ, Jeffreys AS, Datta SK, McDuffie J, Strauss JL, Oddone EZ (2016). A combined patient and provider intervention for management of osteoarthritis in veterans: a randomized clinical trial. Annals of Internal Medicine.

[ref-5] Australian Government Department of Health and Ageing (2015). Telehealth.

[ref-6] Bennell KL, Campbell PK, Egerton T, Metcalf B, Kasza J, Forbes A, Bills C, Gale J, Harris A, Kolt GS, Bunker SJ, Hunter DJ, Brand CA, Hinman RS (2017). Telephone coaching to enhance a home-based physical activity program for knee osteoarthritis: a randomized clinical trial. Arthritis Care & Research.

[ref-7] Blixen CE, Bramstedt KA, Hammel JP, Tilley BC (2004). A pilot study of health education via a nurse-run telephone self-management programme for elderly people with osteoarthritis. Journal of Telemedicine and Telecare.

[ref-8] Brand CA, Harrison C, Tropea J, Hinman RS, Britt H, Bennell K (2014). Management of osteoarthritis in general practice in Australia. Arthritis Care & Research.

[ref-9] Buhrman M, Fältenhag S, Ström L, Andersson G (2004). Controlled trial of Internet-based treatment with telephone support for chronic back pain. Pain.

[ref-10] Burks KJ (2001). Self-management of osteoarthritis: an intervention study.

[ref-11] Carson C (2012). The effective use of effect size indices in institutional research. http://www.keene.edu/ir/effect_size.pdf.

[ref-12] Cottrell MA, Galea OA, O’Leary SP, Hill AJ, Russell TG (2017a). Real-time telerehabilitation for the treatment of musculoskeletal conditions is effective and comparable to standard practice: a systematic review and meta-analysis. Clinical Rehabilitation.

[ref-13] Cottrell MA, Hill AJ, O’Leary SP, Raymer ME, Russell TG (2017b). Patients are willing to use telehealth for the multidisciplinary management of chronic musculoskeletal conditions: a cross-sectional survey. Journal of Telemedicine and Telecare.

[ref-14] Craig P, Dieppe P, Macintyre S, Michie S, Nazareth I, Petticrew M (2008). Developing and evaluating complex interventions: the new medical research council guidance. BMJ.

[ref-15] Cuperus N, Hoogeboom TJ, Kersten CC, Den Broeder AA, Vlieland TPMV, Van Den Ende CHM (2015). Randomized trial of the effectiveness of a non-pharmacological multidisciplinary face-to-face treatment program on daily function compared to a telephone-based treatment program in patients with generalized osteoarthritis. Osteoarthritis and Cartilage.

[ref-16] Damush TM, Weinberger M, Perkins SM, Rao JK, Tierney WM, Qi R, Clark DO (2003). The long-term effects of a self-management program for inner-city primary care patients with acute low back pain. Archives of Internal Medicine.

[ref-17] Dario AB, Moreti Cabral A, Almeida L, Ferreira ML, Refshauge K, Simic M, Pappas E, Ferreira PH (2017). Effectiveness of telehealth-based interventions in the management of non-specific low back pain: a systematic review with meta-analysis. Spine Journal: Official Journal of the North American Spine Society.

[ref-18] De Rezende MU, Hissadomi MI, De Campos GC, Frucchi R, Pailo AF, Pasqualin T, Brito NLR, Santana OFM, Moreira MM, Strutz CG, Matos NBDS, De Camargo OP, Hernandez AJ (2016). One-year results of an educational program on osteoarthritis: a prospective randomized controlled trial in brazil. Geriatric Orthopaedic Surgery & Rehabilitation.

[ref-19] Dinesen B, Nonnecke B, Lindeman D, Toft E, Kidholm K, Jethwani K, Young HM, Spindler H, Oestergaard CU, Southard JA, Gutierrez M, Anderson N, Albert NM, Han JJ, Nesbitt T (2016). Personalized telehealth in the future: a global research agenda. Journal of Medical Internet Research.

[ref-20] Dworkin RH, Turk DC, Farrar JT, Haythornthwaite JA, Jensen MP, Katz NP, Kerns RD, Stucki G, Allen RR, Bellamy N, Carr DB, Chandler J, Cowan P, Dionne R, Galer BS, Hertz S, Jadad AR, Kramer LD, Manning DC, Martin S, McCormick CG, McDermott MP, McGrath P, Quessy S, Rappaport BA, Robbins W, Robinson JP, Rothman M, Royal MA, Simon L, Stauffer JW, Stein W, Tollett J, Wernicke J, Witter J (2005). Core outcome measures for chronic pain clinical trials: IMMPACT recommendations. PAIN.

[ref-21] Egger M, Davey Smith G, Schneider M, Minder C (1997). Bias in meta-analysis detected by a simple, graphical test. BMJ.

[ref-22] Fisher E, Heathcote L, Palermo TM, De C Williams AC, Lau J, Eccleston C (2014). Systematic review and meta-analysis of psychological therapies for children with chronic pain. Journal of Pediatric Psychology.

[ref-23] Gialanella B, Ettori T, Faustini S, Baratti D, Bernocchi P, Comini L, Scalvini S (2017). Home-based telemedicine in patients with chronic neck pain. American Journal of Physical Medicine & Rehabilitation.

[ref-24] Goode AP, Taylor SS, Hastings SN, Stanwyck C, Coffman CJ, Allen KD (2018). Effects of a home-based telephone-supported physical activity program for older adult veterans with chronic low back pain. Physical Therapy.

[ref-25] Graves N, Barnett AG, Halton KA, Veerman JL, Winkler E, Owen N, Reeves MM, Marshall A, Eakin E (2009). Cost-effectiveness of a telephone-delivered intervention for physical activity and diet. PLOS ONE.

[ref-26] Guyatt GH, Oxman AD, Schünemann HJ, Tugwell P, Knottnerus A (2011). GRADE guidelines: a new series of articles in the journal of clinical epidemiology. Journal of Clinical Epidemiology.

[ref-27] Hay SI, Abajobir AA, Abate KH, Abbafati C, Abbas KM, Abd-Allah F, Abdulkader RS, Abdulle AM, Abebo TA, Abera SF, Aboyans V, Abu-Raddad LJ, Ackerman IN, Adedeji IA, Adetokunboh O, Afshin A, Aggarwal R, Agrawal S, Agrawal A, Ahmed MB, Aichour MTE, Aichour AN, Aichour I, Aiyar S, Akinyemiju TF, Akseer N, Lami FHA, Alahdab F, Al-Aly Z, Alam K, Alam N, Alam T, Alasfoor D, Alene KA, Ali R, Alizadeh-Navaei R, Alkaabi JM, Alkerwi A, Alla F, Allebeck P, Allen C, Al-Maskari F, AlMazroa MA, Al-Raddadi R, Alsharif U, Alsowaidi S, Althouse BM, Altirkawi KA, Alvis-Guzman N, Amare AT, Amini E, Ammar W, Amoako YA, Ansha MG, Antonio CAT, Anwari P, Ärnlöv J, Arora M, Artaman A, Aryal KK, Asgedom SW, Atey TM, Atnafu NT, Avila-Burgos L, Avokpaho EFGA, Awasthi A, Awasthi S, Azarpazhooh MR, Azzopardi P, Babalola TK, Bacha U, Badawi A, Balakrishnan K, Bannick MS, Barac A, Barker-Collo SL, Bärnighausen T, Barquera S, Barrero LH, Basu S, Battista R, Battle KE, Baune BT, Bazargan-Hejazi S, Beardsley J, Bedi N, Béjot Y, Bekele BB, Bell ML, Bennett DA, Bennett JR, Bensenor IM, Benson J, Berhane A, Berhe DF, Bernabé E, Betsu BD, Beuran M, Beyene AS, Bhansali A, Bhatt S, Bhutta ZA, Biadgilign S, Bicer BK, Bienhoff K, Bikbov B, Birungi C, Biryukov S, Bisanzio D, Bizuayehu HM, Blyth FM, Boneya DJ, Bose D, Bou-Orm IR, Bourne RRA, Brainin M, Brayne C, Brazinova A, Breitborde NJK, Briant PS, Britton G, Brugha TS, Buchbinder R, Bulto LNB, Bumgarner BR, Butt ZA, Cahuana-Hurtado L, Cameron E, Campos-Nonato IR, Carabin H, Cárdenas R, Carpenter DO, Carrero JJ, Carter A, Carvalho F, Casey D, Castañeda-Orjuela CA, Castle CD, Catalá-López F, Chang J-C, Charlson FJ, Chaturvedi P, Chen H, Chibalabala M, Chibueze CE, Chisumpa VH, Chitheer AA, Chowdhury R, Christopher DJ, Ciobanu LG, Cirillo M, Colombara D, Cooper LT, Cooper C, Cortesi PA, Cortinovis M, Criqui MH, Cromwell EA, Cross M, Crump JA, Dadi AF, Dalal K, Damasceno A, Dandona L, Dandona R, Das Neves J, Davitoiu DV, Davletov K, De Courten B, Leo DD, Steur HD, Defo BK, Degenhardt L, Deiparine S, Dellavalle RP, Deribe K, Deribew A, Jarlais DCD, Dey S, Dharmaratne SD, Dhillon PK, Dicker D, Djalainia S, Do HP, Dokova K, Doku DT, Dorsey ER, Dos Santos KPB, Driscoll TR, Dubey M, Duncan BB, Ebel BE, Echko M, El-Khatib ZZ, Enayati A, Endries AY, Ermakov SP, Erskine HE, Eshetie S, Eshrati B (2017). Global, regional, and national disability-adjusted life-years (DALYs) for 333 diseases and injuries and healthy life expectancy (HALE) for 195 countries and territories, 1990–2016: a systematic analysis for the global burden of disease study 2016. The Lancet.

[ref-28] Higgins J, Green S (2011). http://handbook-5-1.cochrane.org/.

[ref-29] Higgins JPT, Thompson SG, Deeks JJ, Altman DG (2003). Measuring inconsistency in meta-analyses. BMJ.

[ref-30] Hughes SL, Seymour RB, Campbell RT, Desai P, Huber G, Chang HJ (2010). Fit and strong: bolstering maintenance of physical activity among older adults with lower-extremity osteoarthritis. American Journal of Health Behavior.

[ref-31] Hunter DJ (2011). Lower extremity osteoarthritis management needs a paradigm shift. British Journal of Sports Medicine.

[ref-32] Iles R, Taylor NF, Davidson M, O’Halloran P (2011). Telephone coaching can increase activity levels for people with non-chronic low back pain: a randomised trial. Journal of Physiotherapy.

[ref-33] Kroon FPB, Van Der Burg LRA, Buchbinder R, Osborne RH, Johnston RV, Pitt V (2014). Self-management education programmes for osteoarthritis. Cochrane Database of Systematic Reviews.

[ref-34] Li LC, Sayre EC, Xie H, Clayton C, Feehan LM (2017). A community-based physical activity counselling program for people with knee osteoarthritis: feasibility and preliminary efficacy of the track-OA study. JMIR mHealth and uHealth.

[ref-35] Maisiak R, Austin J, Heck L (1996). Health outcomes of two telephone interventions for patients with rheumatoid arthritis or osteoarthritis. Arthritis & Rheumatism.

[ref-36] March L, Amatya B, Osborne RH, Brand C (2010). Developing a minimum standard of care for treating people with osteoarthritis of the hip and knee. Best Practice & Research Clinical Rheumatology.

[ref-37] March ML, Bachmeier CJM (1997). Economics of osteoarthritis: a global perspective. Baillière’s Clinical Rheumatology.

[ref-38] Mazzuca SA, Brandt KD, Katz BP, Chambers M, Byrd D, Hanna M (1997). Effects of self-care education on the health status of inner-city patients with osteoarthritis of the knee. Arthritis & Rheumatism.

[ref-39] McLean S, Sheikh A, Cresswell K, Nurmatov U, Mukherjee M, Hemmi A, Pagliari C (2013). The impact of telehealthcare on the quality and safety of care: a systematic overview. PLOS ONE.

[ref-40] Moher D, Liberati A, Tetzlaff J, Altman DG, PRISMA Group (2009). Preferred reporting items for systematic reviews and meta-analyses: the PRISMA statement. Annals of Internal Medicine.

[ref-41] Nüesch E, Trelle S, Reichenbach S, Rutjes AWS, Tschannen B, Altman DG, Egger M, Jüni P (2010). Small study effects in meta-analyses of osteoarthritis trials: meta-epidemiological study. BMJ.

[ref-42] O’Brien KM, Wiggers J, Williams A, Campbell E, Hodder RK, Wolfenden L, Yoong SL, Robson EK, Haskins R, Kamper SJ, Rissel C, Williams CM (2018). Telephone-based weight loss support for patients with knee osteoarthritis: a pragmatic randomised controlled trial. Osteoarthritis and Cartilage.

[ref-43] Odole AC, Ojo OD (2013). A telephone-based physiotherapy intervention for patients with osteoarthritis of the knee. International Journal of Telerehabilitation.

[ref-44] Rutledge T, Atkinson JH, Chircop-Rollick T, D’Andrea J, Garfin S, Patel S, Penzien DB, Wallace M, Weickgenant AL, Slater M (2018). Randomized controlled trial of telephone-delivered cognitive behavioral therapy versus supportive care for chronic back pain. Clinical Journal of Pain.

[ref-45] Thomas KS, Muir KR, Doherty M, Jones AC, O’Reilly SC, Bassey EJ (2002). Home based exercise programme for knee pain and knee osteoarthritis: randomised controlled trial. BMJ.

[ref-46] Vos T, Abajobir AA, Abate KH, Abbafati C, Abbas KM, Abd-Allah F, Abdulkader RS, Abdulle AM, Abebo TA, Abera SF, Aboyans V, Abu-Raddad LJ, Ackerman IN, Adamu AA, Adetokunboh O, Afarideh M, Afshin A, Agarwal SK, Aggarwal R, Agrawal A, Agrawal S, Ahmadieh H, Ahmed MB, Aichour MTE, Aichour AN, Aichour I, Aiyar S, Akinyemi RO, Akseer N, Lami FHA, Alahdab F, Al-Aly Z, Alam K, Alam N, Alam T, Alasfoor D, Alene KA, Ali R, Alizadeh-Navaei R, Alkerwi A, Alla F, Allebeck P, Allen C, Al-Maskari F, Al-Raddadi R, Alsharif U, Alsowaidi S, Altirkawi KA, Amare AT, Amini E, Ammar W, Amoako YA, Andersen HH, Antonio CAT, Anwari P, Ärnlöv J, Artaman A, Aryal KK, Asayesh H, Asgedom SW, Assadi R, Atey TM, Atnafu NT, Atre SR, Avila-Burgos L, Avokphako EFGA, Awasthi A, Bacha U, Badawi A, Balakrishnan K, Banerjee A, Bannick MS, Barac A, Barber RM, Barker-Collo SL, Bärnighausen T, Barquera S, Barregard L, Barrero LH, Basu S, Battista B, Battle KE, Baune BT, Bazargan-Hejazi S, Beardsley J, Bedi N, Beghi E, Béjot Y, Bekele BB, Bell ML, Bennett DA, Bensenor IM, Benson J, Berhane A, Berhe DF, Bernabé E, Betsu BD, Beuran M, Beyene AS, Bhala N, Bhansali A, Bhatt S, Bhutta ZA, Biadgilign S, Bicer BK, Bienhoff K, Bikbov B, Birungi C, Biryukov S, Bisanzio D, Bizuayehu HM, Boneya DJ, Boufous S, Bourne RRA, Brazinova A, Brugha TS, Buchbinder R, Bulto LNB, Bumgarner BR, Butt ZA, Cahuana-Hurtado L, Cameron E, Car M, Carabin H, Carapetis JR, Cárdenas R, Carpenter DO, Carrero JJ, Carter A, Carvalho F, Casey DC, Caso V, Castañeda-Orjuela CA, Castle CD, Catalá-López F, Chang H-Y, Chang J-C, Charlson FJ, Chen H, Chibalabala M, Chibueze CE, Chisumpa VH, Chitheer AA, Christopher DJ, Ciobanu LG, Cirillo M, Colombara D, Cooper C, Cortesi PA, Criqui MH, Crump JA, Dadi AF, Dalal K, Dandona L, Dandona R, Das Neves J, Davitoiu DV, De Courten B, Leo DDD, Defo BK, Degenhardt L, Deiparine S, Dellavalle RP, Deribe K, Jarlais DCD, Dey S, Dharmaratne SD, Dhillon PK, Dicker D, Ding EL, Djalalinia S, Do HP, Dorsey ER, Dos Santos KPB, Douwes-Schultz D, Doyle KE, Driscoll TR, Dubey M, Duncan BB, El-Khatib ZZ, Ellerstrand J, Enayati A, Endries AY, Ermakov SP, Erskine HE, Eshrati B, Eskandarieh S, Esteghamati A, Estep K, Fanuel FBB, Farinha CSES, Faro A, Farzadfar F, Fazeli MS, Feigin VL, Fereshtehnejad S-M, Fernandes JC, Ferrari AJ, Feyissa TR, Filip I (2017). Global, regional, and national incidence, prevalence, and years lived with disability for 328 diseases and injuries for 195 countries, 1990–2016: a systematic analysis for the global burden of disease study 2016. The Lancet.

[ref-47] Vos T, Allen C, Arora M, Barber RM, Bhutta ZA, Brown A, Carter A, Casey DC, Charlson FJ, Chen AZ, Coggeshall M, Cornaby L, Dandona L, Dicker DJ, Dilegge T, Erskine HE, Ferrari AJ, Fitzmaurice C, Fleming T, Forouzanfar MH, Fullman N, Gething PW, Goldberg EM, Graetz N, Haagsma JA, Hay SI, Johnson CO, Kassebaum NJ, Kawashima T, Kemmer L, Khalil IA, Kinfu Y, Kyu HH, Leung J, Liang X, Lim SS, Lopez AD, Lozano R, Marczak L, Mensah GA, Mokdad AH, Naghavi M, Nguyen G, Nsoesie E, Olsen H, Pigott DM, Pinho C, Rankin Z, Reinig N, Salomon JA, Sandar L, Smith A, Stanaway J, Steiner C, Teeple S, Thomas BA, Troeger C, Wagner JA, Wang H, Wanga V, Whiteford HA, Zoeckler L, Abajobir AA, Abate KH, Abbafati C, Abbas KM, Abd-Allah F, Abraham B, Abubakar I, Abu-Raddad LJ, Abu-Rmeileh NME, Ackerman IN, Adebiyi AO, Ademi Z, Adou AK, Afanvi KA, Agardh EE, Agarwal A, Kiadaliri AA, Ahmadieh H, Ajala ON, Akinyemi RO, Akseer N, Al-Aly Z, Alam K, Alam NKM, Aldhahri SF, Alegretti MA, Alemu ZA, Alexander LT, Alhabib S, Ali R, Alkerwi A, Alla F, Allebeck P, Al-Raddadi R, Alsharif U, Altirkawi KA, Alvis-Guzman N, Amare AT, Amberbir A, Amini H, Ammar W, Amrock SM, Andersen HH, Anderson GM, Anderson BO, Antonio CAT, Aregay AF, Ärnlöv J, Artaman A, Asayesh H, Assadi R, Atique S, Avokpaho EFGA, Awasthi A, Quintanilla BPA, Azzopardi P, Bacha U, Badawi A, Balakrishnan K, Banerjee A, Barac A, Barker-Collo SL, Bärnighausen T, Barregard L, Barrero LH, Basu A, Bazargan-Hejazi S, Beghi E, Bell B, Bell ML, Bennett DA, Bensenor IM, Benzian H, Berhane A, Bernabé E, Betsu BD, Beyene AS, Bhala N, Bhatt S, Biadgilign S, Bienhoff K, Bikbov B, Biryukov S, Bisanzio D, Bjertness E, Blore J, Borschmann R, Boufous S, Brainin M, Brazinova A, Breitborde NJK, Brown J, Buchbinder R, Buckle GC, Butt ZA, Calabria B, Campos-Nonato IR, Campuzano JC, Carabin H, Cárdenas R, Carpenter DO, Carrero JJ, Castañeda-Orjuela CA, Rivas JC, Catalá-López F, Chang JC, Chiang PPC, Chibueze CE, Chisumpa VH, Choi JYJ, Chowdhury R, Christensen H, Christopher DJ, Ciobanu LG, Cirillo M, Coates MM, Colquhoun SM, Cooper C, Cortinovis M, Crump JA, Damtew SA, Dandona R, Daoud F, Dargan PI, Das Neves J, Davey G, Davis AC, De Leo D, Degenhardt L, Del Gobbo LC, Dellavalle RP, Deribe K, Deribew A, Derrett S, Jarlais DCD, Dharmaratne SD, Dhillon PK, Diaz-Torné C (2016). Global, regional, and national incidence, prevalence, and years lived with disability for 310 diseases and injuries, 1990–2015: a systematic analysis for the global burden of disease study 2015. The Lancet.

[ref-48] Weinberger M, Tierney WM, Booher P, Katz BP (1989). Can the provision of information to patients with osteoarthritis improve functional status? A randomized, controlled trial. Arthritis & Rheumatism.

[ref-49] World Health Organization (WHO) (2014). Noncommunicable Diseases Country Profiles 2014.

[ref-50] Williams CM, Maher CG, Hancock MJ, McAuley JH, McLachlan AJ, Britt H, Fahridin S, Harrison C, Latimer J (2010). Low back pain and best practice care: a survey of general practice physicians. Archives of Internal Medicine.

[ref-51] Williams A, Wiggers J, O’Brien KM, Wolfenden L, Yoong S, Campbell E, Robson E, McAuley J, Haskins R, Kamper SJ, Williams CM (2016). A randomised controlled trial of a lifestyle behavioural intervention for patients with low back pain, who are overweight or obese: study protocol. BMC Musculoskeletal Disorders.

[ref-52] Williams A, Wiggers J, O’Brien KM, Wolfenden L, Yoong SL, Hodder RK, Lee H, Robson EK, McAuley JH, Haskins R, Kamper SJ, Rissel C, Williams CM (2018). Effectiveness of a healthy lifestyle intervention for chronic low back pain: a randomised controlled trial. Pain.

[ref-53] Williams CM, Williams A, O’Brien K, Wolfenden L, Wiggers J (2014). Preventative care strategies for common risk factors of chronic disease and musculoskeletal pain in patients waiting for specialist consultation. Obesity Research & Clinical Practice.

