# Supplementary material for: Effectiveness of telephone-based interventions for managing osteoarthritis and spinal pain: a systematic review and meta-analysis
Source: PeerJ. 2018 Oct 30;6:e5846. doi: 10.7717/peerj.5846 (PMC6214231; doi:10.7717/peerj.5846)
Supplement: Supplemental Information 2 [file peerj-06-5846-s002.docx]

**Supplemental Table S1.** Search Strategy. Database(s): MEDLINE 1946 to Present with Daily Update

| **#** | **Searches** | **Results** |
| --- | --- | --- |
| 1 | (telephon* or phone*).tw. | 60589 |
| 2 | (telemed* or tele-med* or telehealth* or tele-health* or telecare or tele-care or telehome or tele-home or telemanage* or tele-manage*).tw. | 8237 |
| 3 | (telemonitor* or tele-monitor* or teleconsult* or tele-consult* or telecommunicat* or tele-communicat* or telecounsel* or tele-counsel* or telesupport* or tele-support* or teletherapy or tele-therapy or teleconferenc* or tele-conferenc*).tw. | 5448 |
| 4 | (remote adj3 (communicat* or consult*)).tw. | 411 |
| 5 | (tele* adj nurs*).tw. | 155 |
| 6 | 1 or 2 or 3 or 4 or 5 | 71945 |
| 7 | exp Osteoarthritis/ | 47462 |
| 8 | exp Back Pain/ | 31127 |
| 9 | Neck Pain/ | 5027 |
| 10 | (backache or neckache).tw. | 1965 |
| 11 | Musculoskeletal Pain/ | 1309 |
| 12 | Sciatica/ | 4441 |
| 13 | Neuralgia/ | 9507 |
| 14 | (dorsalgia or cervicalgia).tw. | 124 |
| 15 | ((Cervical Vertebrae or back or knee* or neck or spin* or hip* or lumb* or joint* or musculoske*) adj3 (pain* or ache* or aching or complaint* or stiff* or dysfunction* or disabil* or trauma* or disorder* or injur*)).tw. | 128899 |
| 16 | (osteoarthr* or osteo arthr*).tw. | 44022 |
| 17 | Coxarthr*.tw. | 1600 |
| 18 | 7 or 8 or 9 or 10 or 11 or 12 or 13 or 14 or 15 or 16 or 17 | 206177 |
| 19 | randomized controlled trial.pt. | 414385 |
| 20 | random*.ab. | 705537 |
| 21 | placebo.ab. | 158580 |
| 22 | Clinical Trials as Topic/ | 179503 |
| 23 | trial.ab. | 318798 |
| 24 | 19 or 20 or 21 or 22 or 23 | 1124413 |
| 25 | 6 and 18 and 24 | 366 |
| 26 | (animals not (humans and animals)).sh. | 4040063 |
| **27** | **25 not 26** | **366** |
| 28 | 6 and 18 | 1371 |
| **29** | **28 not 26** | **1344** |
