# Supplementary material for: Effectiveness of telephone-based interventions for managing osteoarthritis and spinal pain: a systematic review and meta-analysis
Source: PeerJ. 2018 Oct 30;6:e5846. doi: 10.7717/peerj.5846 (PMC6214231; doi:10.7717/peerj.5846)
Supplement: Supplemental Information 3 [file peerj-06-5846-s003.docx]

| **Supplemental Table S2.** Any outcomes from a trial that could not be synthesised in meta-analysis | |
| --- | --- |
| **Author (year)** | **Study Results** |
| Allen (2010) | Treatment adherence: In the osteoarthritis self-management group, 1324 intervention calls were made to 172 participants (mean call length, 9.0 minutes; median calls, 9 [IQR, 6 to 10]). In the health education control group, 1539 calls were made to 172 participants (mean call length, 4.9 minutes; median calls, 10 [IQR, 9 to 10]).  Adverse events: No serious study-related adverse events occurred |
| Allen (2016) | Physical activity: MD 1.6, 95%CI 0.3 to 2.9, p=0.017  Healthcare utilisation, %   - Health service use (telephone vs. usual care): physical therapy (1% vs. 3%), knee braces (3% vs. 2%), MOVE! program (5% vs. 1%), orthopaedic visit (3% vs. 4%), and joint injection (5% both) - Change in pain medication use (telephone vs. usual care): 36% vs. 35%   Treatment adherence: Osteoarthritis Intervention participants completed an average of 11.5 phone calls (SD = 4.9) out of 18 planned calls, and the average length of calls was 16.6 minutes (SD =12.4)  Adverse events: 4 study-related adverse events occurred, but none were associated with the osteoarthritis intervention |
| Allen (2017) | Physical activity: MD (95%CI) patient intervention vs. usual care −0.1 (−2.0 to 1.7), p=0.76, provider intervention vs. usual care −0.3 (−2.5 to 1.9), p=0.89, patient-provider intervention vs. usual care 0.1 (−2.1 to 2.4), p=0.90  Healthcare utilisation, % (patient intervention vs. provider intervention vs. patient-provider intervention vs. usual care)   - Health service use: physical therapy (13% vs. 13% vs. 9% vs. 8%), knee braces (9% vs. 6% vs. 8% vs. 6%), joint injection (24% vs, 23% vs. 17% vs. 19%), joint replacement surgery (4% vs. 2% vs. 4% vs. 3%) - New pain medication: 28% vs. 33% vs. 39% vs. 29%   Treatment adherence: Participants in the patient interventions completed an average of 7.8 (SD, 4.6) telephone calls of a possible 18. Participants in the patient–provider interventions completed an average of 8.4 (SD, 4.3) telephone calls of a possible 18.  Adverse events: No study-related adverse events occurred |
| Bennell (2017) | Physical activity: MD in change score -1.7, 95%CI -7.1 to 3.8  Healthcare utilisation, n (%)  Telephone plus comprehensive face-to-face intervention vs. face-to-face intervention alone   - Health service use: 61 (97%) vs. 57 (92%) - Pain medication use: 39 (64%) vs. 41 (72%)   Treatment adherence (home exercise): MD in change score 5, 95%CI -7 to 17  Treatment adherence (calls and physiotherapy sessions): Telephone calls (mean (SD)): 5.4 (2.0) ranging from 0 to 9, with the mean (SD) call duration being 39.6 (11.5) and 24.1 (10.2) minutes for initial and subsequent calls. Physiotherapy sessions: (mean (SD)): 4.4 (1.2) for telephone plus comprehensive face-to-face and 4.3 (1.4) for face-to-face (p>0.05).  Health-related QoL: MD in change score 0.0, 95%CI -0.1 to 0.0  Subjective improvement: OR 2.1, 95%CI 1.0 to 4.4  Adverse events: were mild (mostly transient increased knee pain) and reported by approximately one-third of participants during the intervention, but were infrequent during follow-up. |
| Burks (2001) | Disability: no significant difference by treatment group (p=0.57) |
| Cuperus (2015) | Self-efficacy: MD in change score between groups 0.84, 95%CI -0.44 to 2.12  Physical activity: OR 1.55, 95%CI 0.65 to 3.74  Treatment adherence: The mean number of sessions participants attended was 6.6 (out of 7) in the face-to-face group and 5.5 (out of 6) in the telephone based treatment group  Fear avoidance: MD in change score between groups -0.48, 95%CI -2.52 to 1.56  Adverse events: No adverse events related to the treatment programs were reported |
| Damush (2003) | Average treatment effect between groups  Physical activity (minutes): 42.00, 95%CI 0.63 to 83.37, p=0.047  Fear avoidance: -2.35, 95%CI -3.96 to -0.74, p=0.005 |
| Gialanella (2017) | Treatment adherence (telephone plus comprehensive face-to-face intervention vs. face-to-face intervention alone): home exercises, % patients per category: null/occasional sessions/2-4 sessions per week/≥5 sessions per week = 2.1/10.6/44.7/42.6 vs. 25.6/8.5/36.1/29.8, p=0.012  Adverse events: “We did not register any adverse effects during the study period” |
| Goode  (2018) | Disability (between-group changes, mean (95%CI)): Physical activity vs. wait-list: -4.10 (-6.85 to -1.34), Physical activity + CBT-pain vs. wait-list: -1.99 (-4.85 to 0.86).  Treatment adherence: Among all participants who initiated the physical activity-only or physical activity + CBT-pain program, the average numbers of intervention calls completed (out of a possible total of 13) were 10.0 (SD = 1.46) for the physical activity group and 8.8 (SD = 2.37) for the physical activity + CBT-pain group.  Adverse events: “There were no study-related adverse events” |
| Hughes (2010) | Negotiation vs. maintenance, coefficient (z score), p-value  Pain intensity: -0.618 (-1.161), p=0.123  Disability: -0.785 (-0.49), p=0.313  Psychological symptoms: 0.013 (0.06), p=0.952  Weight loss: -0.351 (-0.44), p=0.329  Physical activity: 0.109 (0.13), p=0.449  Adverse events: No adverse health outcomes were reported by participants |
| Iles (2011) | Recovery: MD 3.4, 95%CI 1.1 to 5.7 |
| Li (2017) | Physical activity (telephone plus face-to-face vs. waitlist control): mean (SD) 64.2 (70.5) vs. 56.0 (60.1)  Adverse events: “No adverse event associated with the intervention was reported by participants during the study” |
| Maisiak (1996) | 1. Telephone vs. usual care  Psychological symptoms: Treatment effect size 0.15, 95%CI -0.32 to 0.43  Healthcare utilisation (health service use): 2.71 vs. 4.28, p<0.01  2. Telephone vs. attention control  Psychological symptoms: Treatment effect size -0.06, 95%CI -0.45 to 0.31  Healthcare utilisation (health service use): 2.71 vs. 3.88, p>0.01 |
| Mazzuca (1997) | Telephone vs. attention control  Pain intensity: mean (SD) 5.84 (3.19) vs. 6.74 (2.78), p=0.100  Disability: mean (SD) 1.26 (0.65) vs. 1.29 (0.70), p=0.135  Health-related QoL: mean (SD) 0.56 (0.08) vs. 0.57 (0.08), p=0.569 |
| O’Brien (2018) | MD (95%CI)  Pain: -0.6 (-1.4 to 0.2)  Disability: -2.9 (-8.5, 2.7)  Psychological symptoms: 1.2 (-1.4, 3.9)  Weight loss: -0.2 (-1.0, 0.6)  Physical activity: 5.3 (-142.8, 153.4)  Healthcare utilisation, OR (95%CI)   - Health service use: 0.54 (0.19, 1.52) - Pain medication use: 0.63 (0.24, 1.64)   Subjective improvement: 0.3 (-0.7, 1.2)  Fear avoidance: -3.7 (-6.4, -1.1)  Treatment adherence: 95% of participants in the intervention group commenced GHS coaching calls, 51% received at least two calls [median calls two; interquartile range (IQR): 1-8.5]. Twenty participants (34%) in the intervention group received six or more coaching calls.  Adverse events: There were 35 adverse events reported in each group. The proportion of participants reporting an adverse event was not different between groups |
| Odole (2013) | Telephone vs. face-to-face intervention  Mean (SD), t-value, p-value  Pain intensity: 22.40 (13.76) vs. 18.84 (15.955), t=0.844, p=0.40  Disability: -83.70 (10.26) vs. -84.87 (10.79), t=0.391, p=0.70  Psychological symptoms: -71.96 (7.55) vs. -71.40 (8.23), t=0.025, p=0.80 |
| de Rezende (2016) | Telephone plus comprehensive face-to-face intervention vs. face-to-face intervention alone  Group 1  Weight loss: mean (SD) 30.1 (5.4) vs. 31.8 (13.1)  Treatment adherence (attended lectures and/or retrieved material): 86% vs. 100%  Group 2  Weight loss: mean (SD) 32.8 (6.8) vs. 32.4 (5.3)  Treatment adherence: 93% vs. 89%  Group 3  Weight loss mean (SD) 29.7 (4.3) vs. 32 (5.8)  Treatment adherence: 93% vs. 93%  Group 4  Weight loss: mean (SD) 30.5 (6.2) vs. 31.9 (5.5)  Treatment adherence: 90% vs. 100%  Adverse events: There were no adverse events in any group |
| Rutledge (2018) | Subjective improvement (n, %): telephone 30.4 (31) vs. attention control 22.7 (18.5)  Treatment adherence: Participants completed >90% of planned telephone treatment sessions in both conditions. Supportive care participants completed an average of 10.8 (0.76) out of a maximum of 11 telephone sessions, whereas CBT participants completed an average of 10.0 (2.2) of 11 sessions  Adverse events: No adverse events were reported by participants during the trial |
| Thomas (2002) | Pain intensity: MD -0.16, 95%CI -0.6 to 0.3  Treatment adherence: Self-reported adherence to the exercise programme was crudely graded as high (n=128), medium (n=32), or low (n=307) |
| Weinberger (1989) | Pain intensity (mean): telephone only 5.76, telephone plus clinic 6.04, clinic only 6.24, and usual care 6.58  Disability (mean): telephone only 2.47, telephone plus clinic 2.67, clinic only 2.97, and usual care 2.65  Psychological symptoms (mean): telephone only 2.94, telephone plus clinic 2.88, clinic only 3.27, and usual care 3.04 |
| Williams 2018 | MD (95%CI)  Weight loss: 0.6 (0.0, 1.2)  Physical activity: -99.3 (-260.2, 61.5)  Healthcare utilisation, OR (95%CI)   - Health service use: 0.73 (0.33, 1.65) - Pain medication use: 0.54 (0.20, 1.44)   Subjective improvement: -0.6 (-1.3, 0.2)  Fear avoidance: 1.0 (-1.4, 3.5)  Treatment adherence: 37 participants attended the single consultation with the study physiotherapist. 76 participants commenced GHS coaching calls (received at least one call), 38 (48.1%) participants received at least 3 calls (median 3; 14 interquartile range: 1 to 9), and 33 (41.8%) participants receiving 6 or more calls. The mean number calls conducted with participants was 5.1 (SD 4.5). Twenty-three participants (29.1%) attended the clinical consult and received 6 or more calls.  Adverse events: The proportion of participants reporting an adverse event was not different between groups; 41% (n=32) and 56% (n=45) for the intervention and control group respectively |

CBT=cognitive behaviour therapy, GHS=Get Healthy Service, QoL=quality of life
