# Supplementary material for: Effectiveness of telephone-based interventions for managing osteoarthritis and spinal pain: a systematic review and meta-analysis
Source: PeerJ. 2018 Oct 30;6:e5846. doi: 10.7717/peerj.5846 (PMC6214231; doi:10.7717/peerj.5846)
Supplement: Supplemental Information 4 — Notes: Green, low risk; yellow, unclear risk; red, high risk. [file peerj-06-5846-s004.docx]

**Supplemental Figure S1.** Risk of bias summary showing review authors’ judgments about each risk of bias domain in trials included the review. Trials are listed alphabetically by author name*


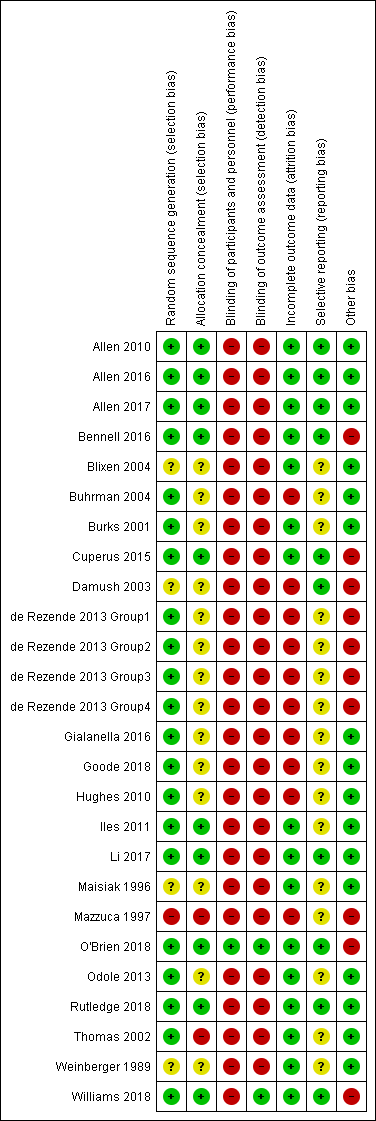


Notes: Green, low risk; yellow, unclear risk; red, high risk

*In four studies (Allen 2016, Allen 2017, Hughes 2010, Rezende 2013) for blinding of outcome assessment (detection bias) risk of bias was low for objectively measured weight.
