# Supplementary material for: Effectiveness of telephone-based interventions for managing osteoarthritis and spinal pain: a systematic review and meta-analysis
Source: PeerJ. 2018 Oct 30;6:e5846. doi: 10.7717/peerj.5846 (PMC6214231; doi:10.7717/peerj.5846)

**Supplemental Figure S2.** Forest plots of main meta-analyses findings for comparison telephone-based interventions (with educational materials) versus usual care

Forest plot of outcome: Pain intensity


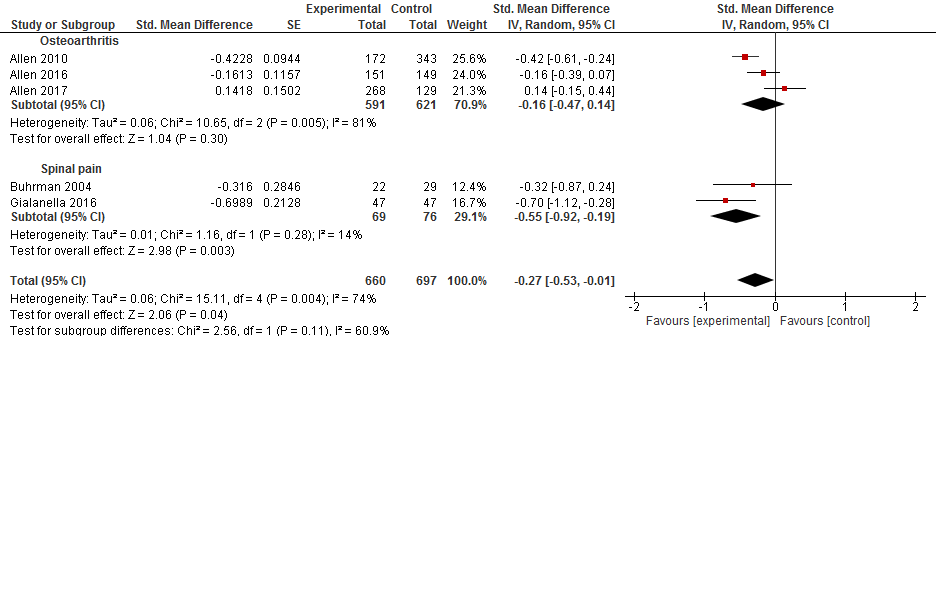


Forest plot of outcome: Disability


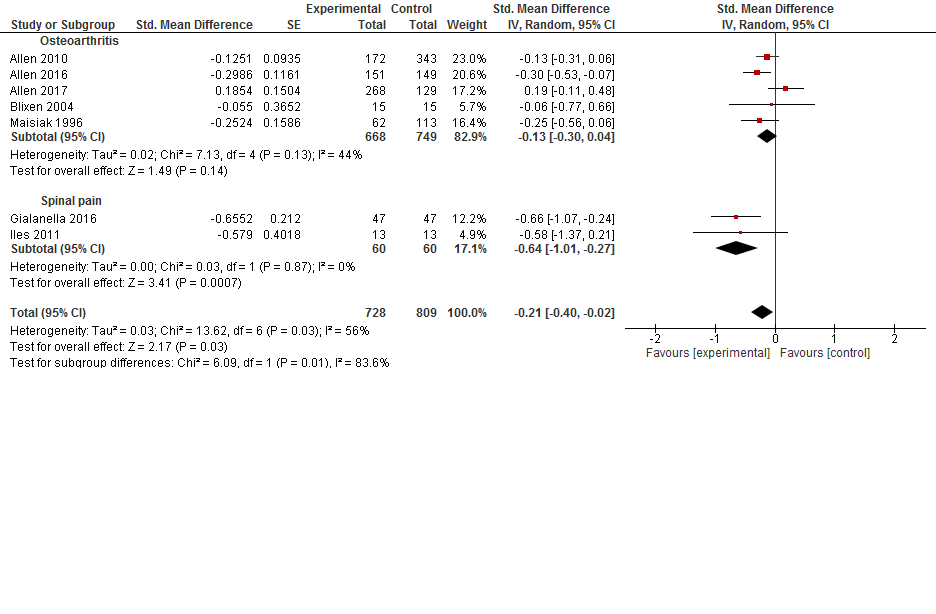


Forest plot of outcome: Psychological symptoms


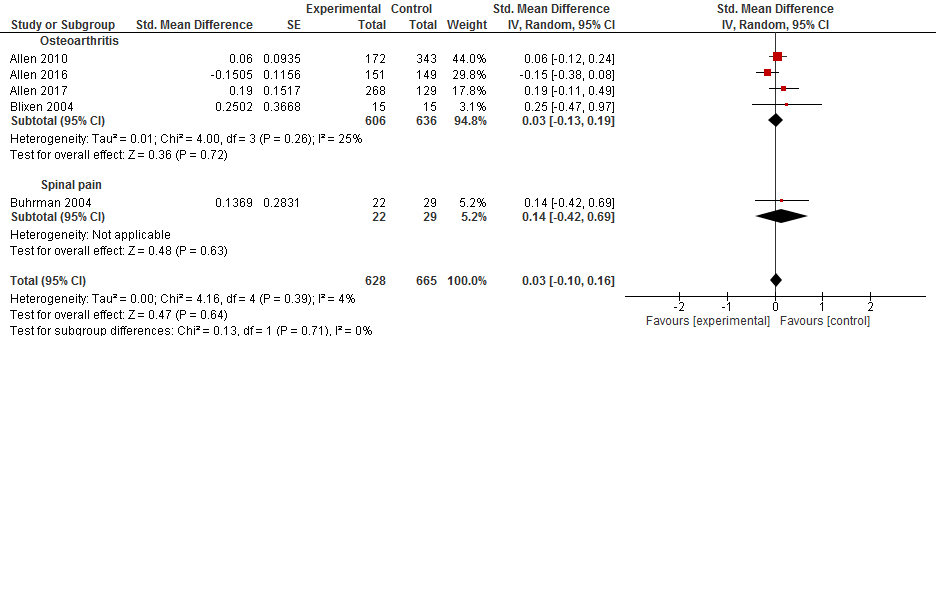


Forest plot of outcome: Self-efficacy


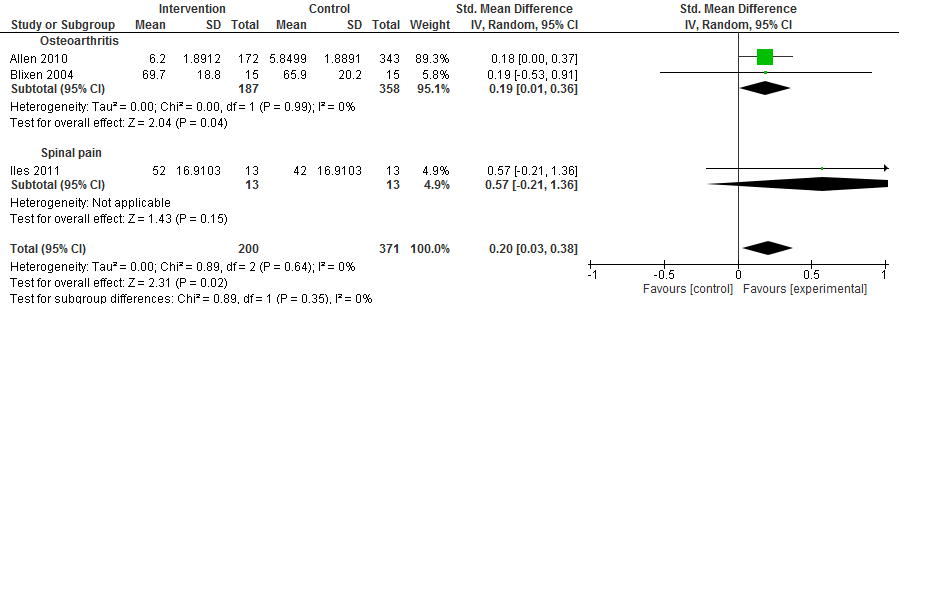


Forest plot of outcome: Weight loss


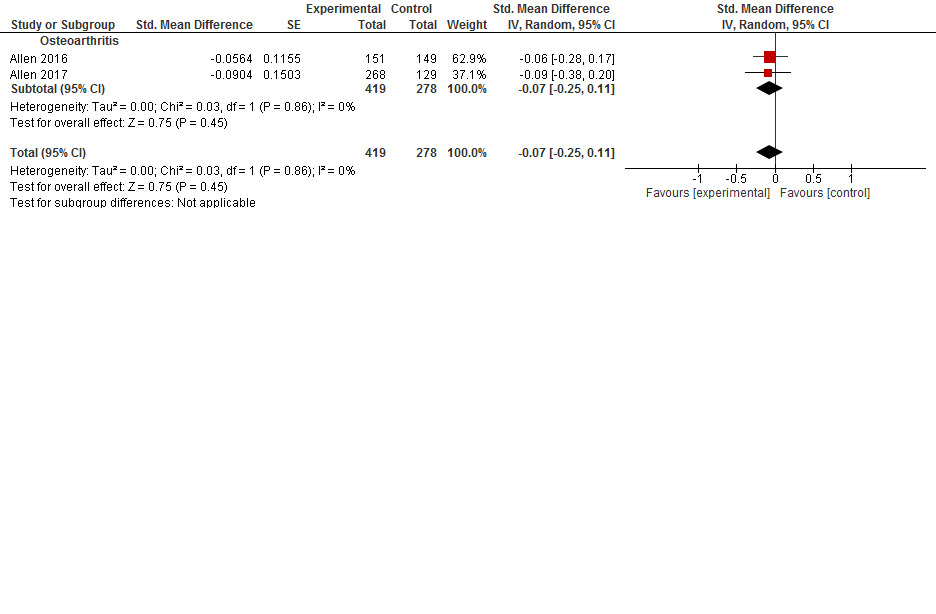

Supplement: Supplemental Information 5 [file peerj-06-5846-s005.docx]
