# Supplementary material for: Effectiveness of telephone-based interventions for managing osteoarthritis and spinal pain: a systematic review and meta-analysis
Source: PeerJ. 2018 Oct 30;6:e5846. doi: 10.7717/peerj.5846 (PMC6214231; doi:10.7717/peerj.5846)

**Supplemental Figure S4.** Forest plots of main meta-analyses findings for comparison telephone plus face-to-face interventions versus usual care

Forest plot of outcome: Pain intensity


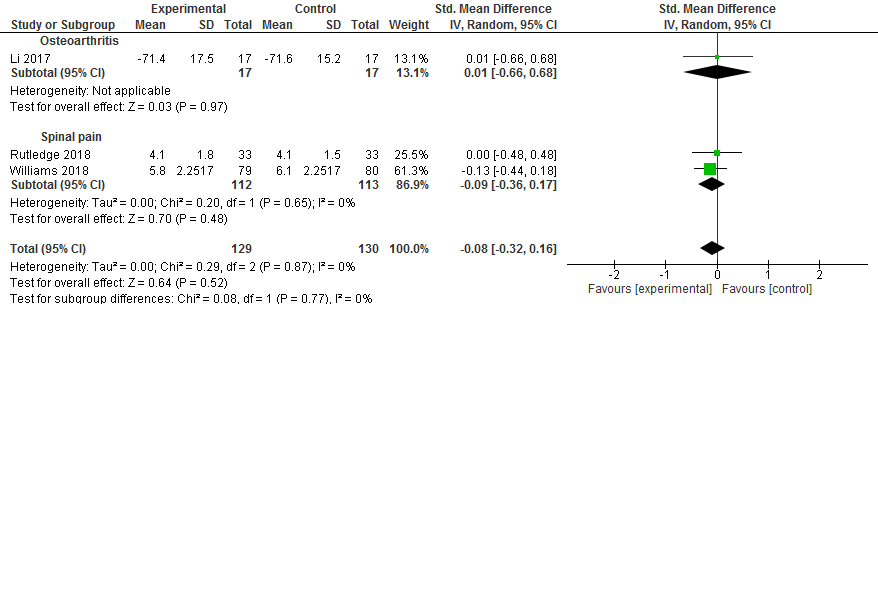


Forest plot of outcome: Disability


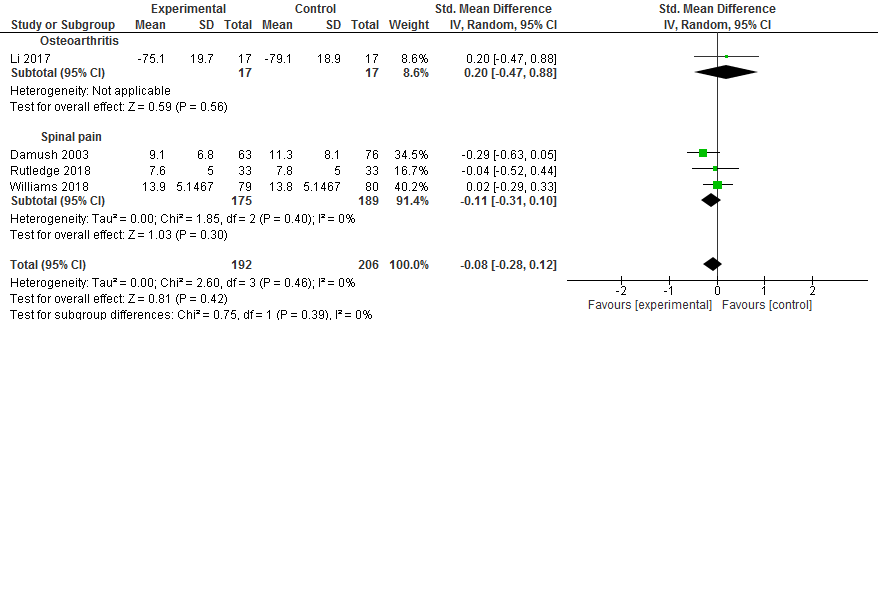


Forest plot of outcome: Psychological symptoms


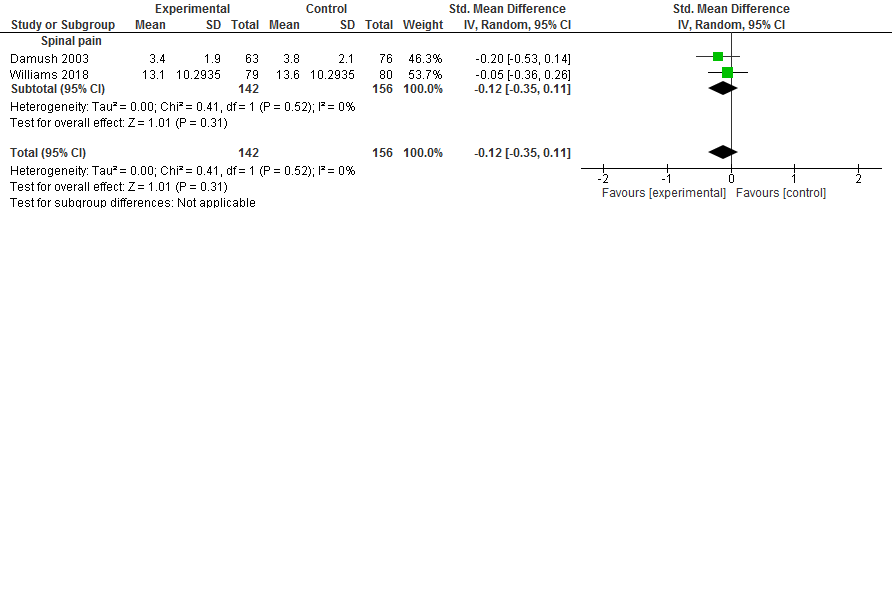

Supplement: Supplemental Information 7 [file peerj-06-5846-s007.docx]
