# Supplementary material for: Effectiveness of telephone-based interventions for managing osteoarthritis and spinal pain: a systematic review and meta-analysis
Source: PeerJ. 2018 Oct 30;6:e5846. doi: 10.7717/peerj.5846 (PMC6214231; doi:10.7717/peerj.5846)

**Supplemental Figure S5.** Forest plots of disability outcome subgroup analysis comparison telephone plus face-to-face interventions versus usual care

Subgroup analysis: Spinal pain duration – chronic


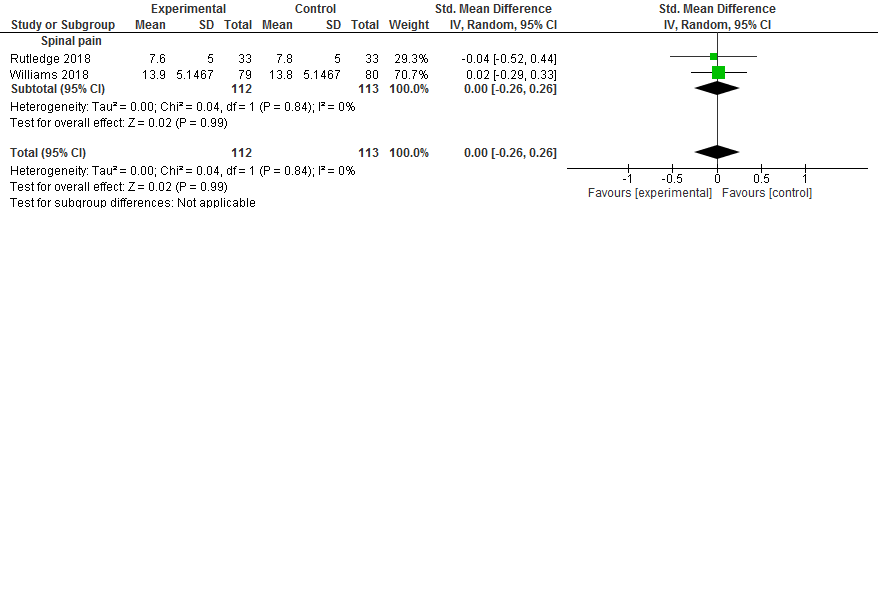

Supplement: Supplemental Information 8 [file peerj-06-5846-s008.docx]
