# Supplementary material for: Effectiveness of telephone-based interventions for managing osteoarthritis and spinal pain: a systematic review and meta-analysis
Source: PeerJ. 2018 Oct 30;6:e5846. doi: 10.7717/peerj.5846 (PMC6214231; doi:10.7717/peerj.5846)
Supplement: Supplemental Information 9 [file peerj-06-5846-s009.docx]

**Supplemental Figure S6.** Forest plots of main meta-analyses findings for comparison telephone plus comprehensive face-to-face interventions versus face-to-face interventions alone

Forest plot of outcome: Pain intensity


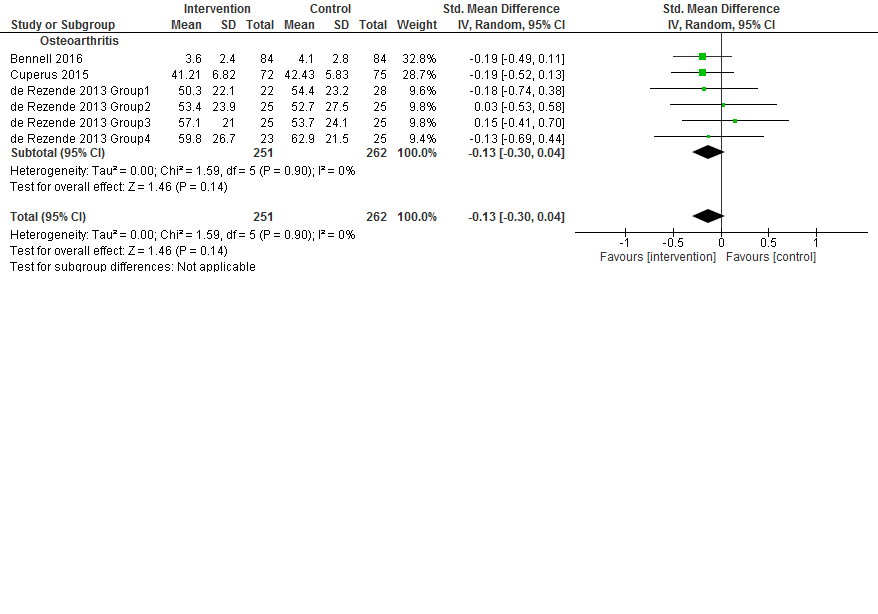


Forest plot of outcome: Disability


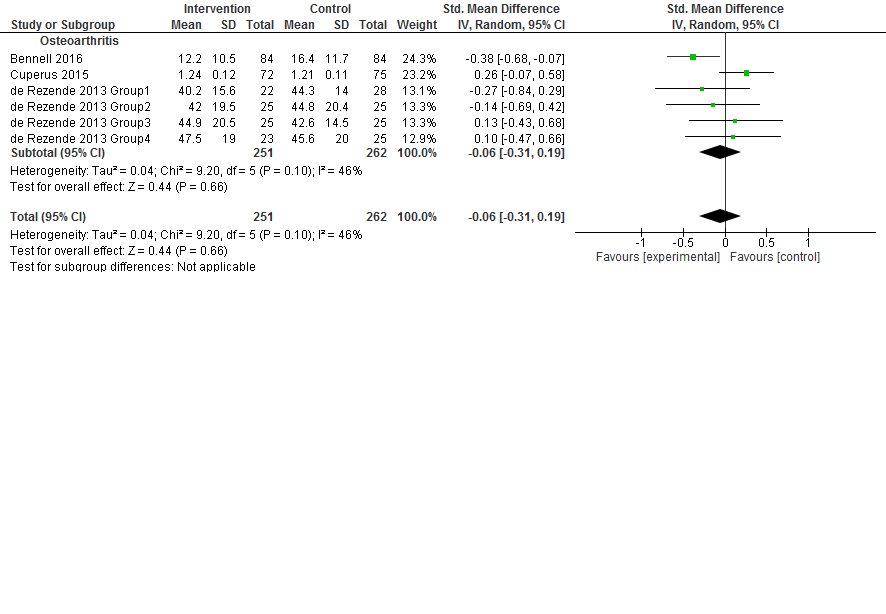


Forest plot of outcome: Psychological symptoms

**
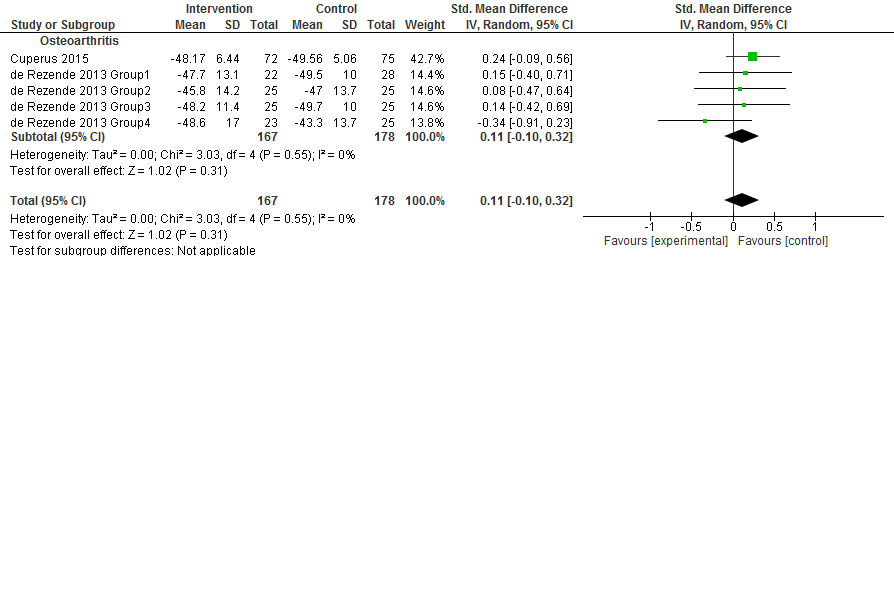
**
