# Supplementary material for: Effectiveness of telephone-based interventions for managing osteoarthritis and spinal pain: a systematic review and meta-analysis
Source: PeerJ. 2018 Oct 30;6:e5846. doi: 10.7717/peerj.5846 (PMC6214231; doi:10.7717/peerj.5846)

**Supplemental Figure S8.** Forest plots of pain intensity outcome sensitivity analysis for comparison telephone-based interventions (with educational material) versus usual care

Sensitivity analysis: Excluding small trials


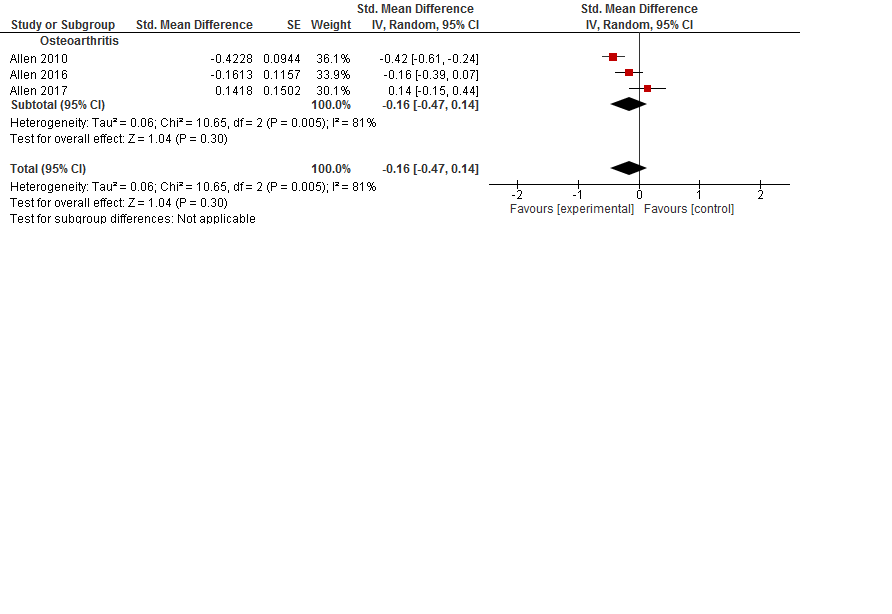

Supplement: Supplemental Information 11 [file peerj-06-5846-s011.docx]
